# Cortical versus hippocampal network dysfunction in a human brain assembloid model of epilepsy and intellectual disability

**DOI:** 10.1016/j.celrep.2025.116217

**Published:** 2025-09-08

**Authors:** Colin M. McCrimmon, Daniel Toker, Marie Pahos, Qing Cao, Kevin Lozano, Jack J. Lin, Jack M. Parent, Andrew Tidball, Jie Zheng, László Molnár, Istvan Mody, Bennett G. Novitch, Ranmal A. Samarasinghe

**Affiliations:** 1 Department of Neurology, University of California, Los Angeles, David Geffen School of Medicine, Los Angeles, CA 90095, USA; 2 Department of Neurology, University of California, Davis, School of Medicine, Sacramento, CA 95817, USA; 3 Center for Mind and Brain, University of California, Davis, Davis, CA 95618, USA; 4 Department of Neurology, University of Michigan School of Medicine, Ann Arbor, MI 48109, USA; 5 Michigan Neuroscience Institute, University of Michigan, Ann Arbor, MI 48109, USA; 6 VA Ann Arbor Healthcare System, Ann Arbor, MI 48105, USA; 7 Department of Biomedical Engineering, University of California, Davis, Davis, CA 95616, USA; 8 Department of Neurological Surgery, University of California, Davis, School of Medicine, Sacramento, CA 95817, USA; 9 Department of Electrical Engineering, Sapientia Hungarian University of Transylvania, Târgu-Mures‚/Corunca 540485, Romania; 10 Department of Physiology, University of California, Los Angeles, David Geffen School of Medicine, Los Angeles, CA 90095, USA; 11 Department of Neurobiology, University of California, Los Angeles, Los Angeles, CA 90095, USA; 12 Eli and Edythe Broad Center for Regenerative Medicine and Stem Cell Research, University of California, Los Angeles, Los Angeles, CA 90095, USA; 13 Intellectual Development and Disabilities Research Center, University of California, Los Angeles, Los Angeles, CA 90095, USA

## Abstract

Neurodevelopmental disorders often impair multiple cognitive domains. For instance, a genetic epilepsy syndrome might cause seizures due to cortical hyperexcitability and present with memory impairments arising from hippocampal dysfunction. This study examines how a single disorder differentially affects distinct brain regions using induced pluripotent stem cell (iPSC)-derived cortical- and hippocampal-ganglionic eminence assembloids to model developmental and epileptic encephalopathy 13, a condition arising from gain-of-function mutations in the *SCN8A* gene encoding the sodium channel Nav1.6. While cortical assembloids showed network hyperexcitability akin to epileptogenic tissue, hippocampal assembloids did not, and instead displayed network dysregulation patterns similar to *in vivo* hippocampal recordings from epilepsy patients. Predictive computational modeling, immunohistochemistry, and single-nucleus RNA sequencing revealed changes in excitatory and inhibitory neuron organization that were specific to hippocampal assembloids. These findings highlight the unique impacts of a single pathogenic variant across brain regions and establish hippocampal assembloids as a platform for studying neurodevelopmental disorders.

**In brief:**

McCrimmon and Toker et al. use region-specific human brain assembloids to model how a genetic epileptic encephalopathy (developmental and epileptic encephalopathy 13) caused by SCN8A variants differentially affects cortical and hippocampal circuits. Their findings uncover distinct, region-specific pathophysiology and establish hippocampal assembloids as a platform for disease modeling.

## Graphical Abstract

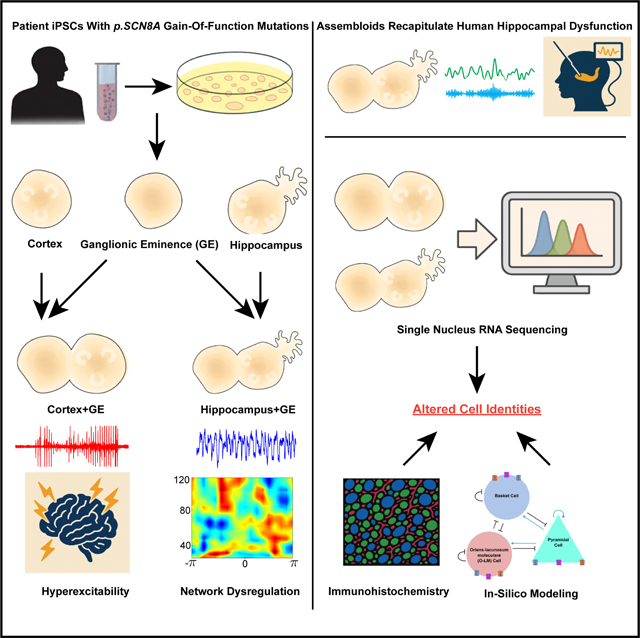

## INTRODUCTION

Human neurodevelopmental disorders are often characterized by complex phenotypes involving neural network dysfunction across multiple brain regions. Despite this, our understanding of how a single perturbation can uniquely impact brain development and network function in different regions remains limited. This knowledge is crucial for developing therapies to treat the various comorbidities associated with these disorders. For instance, in genetic epilepsy syndromes, seizures may result from cortical hyperexcitability, while equally debilitating impairments in learning and memory may arise primarily from distinct cellular dysfunction in the hippocampus. Disentangling regional contributions has proven difficult due to the complexity of regional interactions seen in whole-brain studies, which can obscure results, and the use of animal models that frequently fail to replicate the full range of human disease phenotypes^1^ or present unique, non-human phenotypes. In addition, a historical assumption in epilepsy is that seizures themselves result in widespread neural injury, thereby causing comorbidities such as intellectual disability. In this case, seizure prevention alone would be considered both necessary and sufficient to achieve disease remission. However, this concept has been recently challenged by the observation that cognitive disturbances can present in genetic epilepsies prior to seizures and/or in the absence of significant seizure burden,^2,3^ thus suggesting the need for alternative therapeutic approaches.

To address these questions, we developed human pluripotent stem cell (iPSC)-derived brain-region-specific organoids and assembloids (fusions of different organoid subtypes) of developmental and epileptic encephalopathy 13 (DEE-13). This is a condition caused by gain-of-function mutations in the *SCN8A* gene encoding the sodium channel Nav1.6. DEE-13, like other DEEs, typically manifests as early-onset epilepsy and intellectual disability. These symptoms are believed to result from separate developmental perturbations, but the mechanisms driving epilepsy and intellectual disability are unclear.^2^ Our assembloids replicate key features of network-wide neural electrodynamics, such as oscillatory field potentials with spectral peaks at frequencies observed in the human brain *in vivo*, as well as certain pathological dynamics associated with clinical disease.^4^ However, the extent to which aberrant neural activities in organoids correlate with functional changes in humans *in vivo* remains uncertain.

We accordingly generated human cortical and hippocampal assembloids from DEE-13 patient-derived iPSCs, as many seizures in DEE-13 originate in or involve the cortex, while the hippocampus, distinct from the neocortex, plays a crucial role in learning and memory.^5,6^ Since the electrodynamics of hippocampal assembloids have not been characterized previously, we compared network activity in our assembloids with *in vivo* human electrophysiological recordings from healthy and epileptic hippocampi. This comparison revealed a striking similarity in activity profiles, validating our modeling approach. We identified both shared and unique cellular and transcriptomic pathways between these regions and correlated these with unique regional network electrodynamics. These findings indicate distinct causal network pathologies in the cortex and hippocampus underlying epilepsy versus cognitive impairments in DEE-13 and suggest the need for new and more highly targeted therapeutic interventions.

## RESULTS

### Generation of integrated neural assembloids

**Figure 1.**
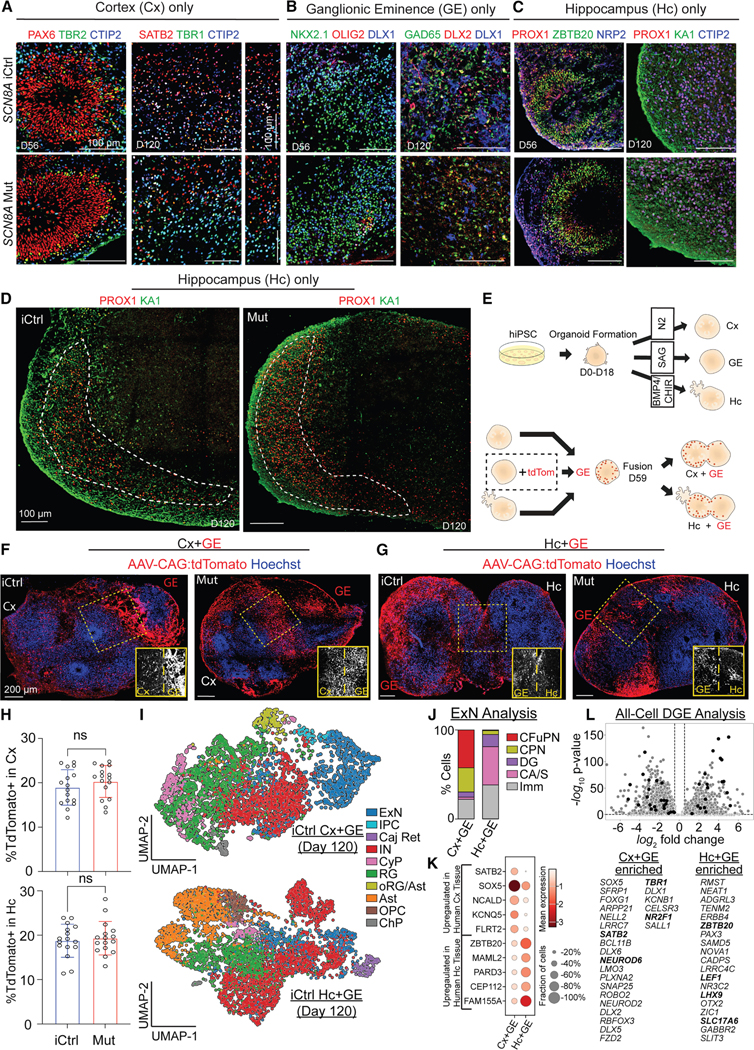
Generation and characterization of cortical and hippocampal assembloids (A) IHC analysis of SCN8A patient iPSC-derived isogenic control (iCtrl) and mutant (Mut) unfused Cx organoids at specified ages shows similar formation and layered segregation of neural progenitors (PAX6 and TBR2) and deep/superficial neurons (CTIP2, TBR1, and SATB2). (B) Organoids from both conditions display comparable levels of GE progenitor and migratory interneuron markers (NKX2–1, OLIG2, DLX1, and DLX2) and GABAergic interneuron marker GAD65 by day 120. (C) Analysis of p.SCN8A iCtrl and Mut unfused hippocampal organoids at days 56 and 120 reveals the expression of hippocampal neuron markers (ZBTBT20 and NRP2) and dentate granule cells (PROX1), with CA3 and dentate granule layers at day 120. (D) PROX1-expressing dentate granule cells are segregated from KA1-expressing CA3 region. (E) Schematic of organoid generation, patterning, and fusion to create assembloids, with GE organoids treated with AAV1-CAG virus prior to fusion. (F and G) At day 84 *in vitro*, both iCtrl and Mut Cx+GE (F) and Hc+GE (G) show similar migration of GE-derived Tdtomato-labeled cells. (H) Quantification shows no significant differences; *n* = 4 independently generated assembloids per genotype, four sections per assembloid with ≥ 1,188 cells/sample for Cx+GE and 790 cells/sample for Hc+GE. Individual datapoints along with their mean ± SD are shown. *p* = 0.6756 for Cx+GE and *p* = 0.3254 for Hc+GE; Student’s t test. (I) UMAP of day 120 iCtrl Cx+GE and iCtrl Hc+GE assembloids with cell types (ExN, excitatory neurons; IPC, intermediate progenitors; Caj Ret, Caja-Retzius; IN, inhibitory neurons; CyP, cycling progenitors; RG, radial glia; oRG, outer radial glia; Ast, astrocytes; OPC, oligodendrocyte precursors; and ChP, choroid plexus). (J) Analysis of the ExN group demonstrates that Cx+GE and Hc+GE assembloids contain excitatory subtypes (CFuPN, corticofugal projection neurons; CPN, callosal projection neurons; DG, dentate granule-like; CA/S, cornu ammonis/subiculum-like; and Imm, immature) in the expected proportions. See Table S2 for gene expression differences. (K) Compared to publicly available *ex vivo* human datasets, Cx+GE ExNs express gene markers that are differentially upregulated in the cerebral cortex neurons, and Hc+GE ExNs express gene markers that are differentially upregulated in the hippocampal neurons. (L) Volcano plot and gene list (in order of statistical significance) highlight unique genes distinguishing Cx+GE from Hc+GE assembloids across all cell types (not just ExNs) with published discriminating genes indicated in bold. Scale bars: 100 μm in (A)–(D) and 200 μm in (F) and (G).

We generated iPSC-derived organoids from a DEE-13 patient, patient 1 (P1), with a gain-of-function variant (p.R1872>L) in the *SCN8A* gene, associated with refractory seizures and intellectual disability.^7^ Organoids from P1 (Mut) and a matched CRISPR-corrected control line (iCtrl) were directed toward cortical, hippocampal, or ganglionic eminence (GE) fates. By day 56, cortical organoids expressed PAX6 (radial glial progenitors), TBR2 (intermediate progenitors), and CTIP2 (deep cortical layers)^8–10^ (Figures 1A and S1D). By day 120, they expressed SATB2 (superficial layers), TBR1, and CTIP2^9,11^ (Figures 1A and S1I). GE-directed organoids expressed NKX2–1, OLIG2 (GE progenitors), and DLX1 (migratory interneurons) by day 56^12–14^ (Figures 1B and S1D) and glutamic acid decarboxylase-65 (GAD65) (GABAergic neurons) by day 120^15^ (Figure 1B). Hippocampal fate organoids expressed NRP2 (hippocampal progenitors), PROX1 (dentate granule cells), and ZBTB20 (hippocampal primordium) at day 56^16–18^ (Figures 1C and S1D) and KA1 (CA3 pyramidal cells) and CTIP2 (CA1/CA2 granule cells) at day 120^19,20^ (Figures 1C, 1D, S1C, and S1H).

On day 56, GE organoids were fused with the cortical (Cx) or hippocampal (Hc) organoids (Figure 1E). A subset of Cx, Hc, and GE organoids were fused at day 59, with the GE organoids pre-treated with adeno-associated virus (AAV) CAG:tdTomato on day 56. These fusions showed widespread migration of tdTomato + cells into Cx (Figure 1F) and Hc (Figure 1G) compartments post-fusion. By 2 weeks post-fusion, 18% of the cells in both compartments were tdTomato+ with no difference between Mut and iCtrls (Figure 1H). By day 120, Cx+GE assembloids expressed BRN2 (POU3F2, superficial cortical layer),^21,22^ GAD65 (inhibitory IN), and GFAP (astrocytes)^23^ (Figure S1A). Hc+GE assembloids expressed MYT1L (neurons) and GFAP and maintained distinct dentate granule-like (PROX1+) and CA3-like (KA1+) regions (Figure S1B).

Single-nucleus RNA sequencing (snRNAseq) of iCtrl Cx+GE and Hc+GE assembloids at day 120 revealed rich cell-type diversity (Figure 1I) and widespread p.SCN8A expression. Cx+GE assembloids expressed markers of corticofugal and callosal projection neurons (CFuPNs and CPNs, respectively), while Hc+GE expressed dentate gyrus (DG) and CA/subiculum (CA/S) markers (Figure 1J). Moreover, the Cx+GE neurons exhibited substantial gene expression overlap with the cerebral cortex neurons from *ex vivo* human datasets and were distinct from the Hc+GE neurons, which exhibited substantial gene expression overlap with *ex vivo* hippocampal neurons^24^ (Figure 1K). When looking at all cells, not just neurons, Cx+GE were enriched for cortical markers *SATB2*, *NEUROD6*, and *TBR1*, while Hc+GE were enriched for hippocampal markers *ZBTB20*, *LEF1*, and *LHX9* (Figure 1L), and these findings are consistent with prior studies on the developing mammalian cortex and hippocampus.^25,26^

### DEE-13 cortical but not hippocampal assembloids are hyperexcitable

**Figure 2.**
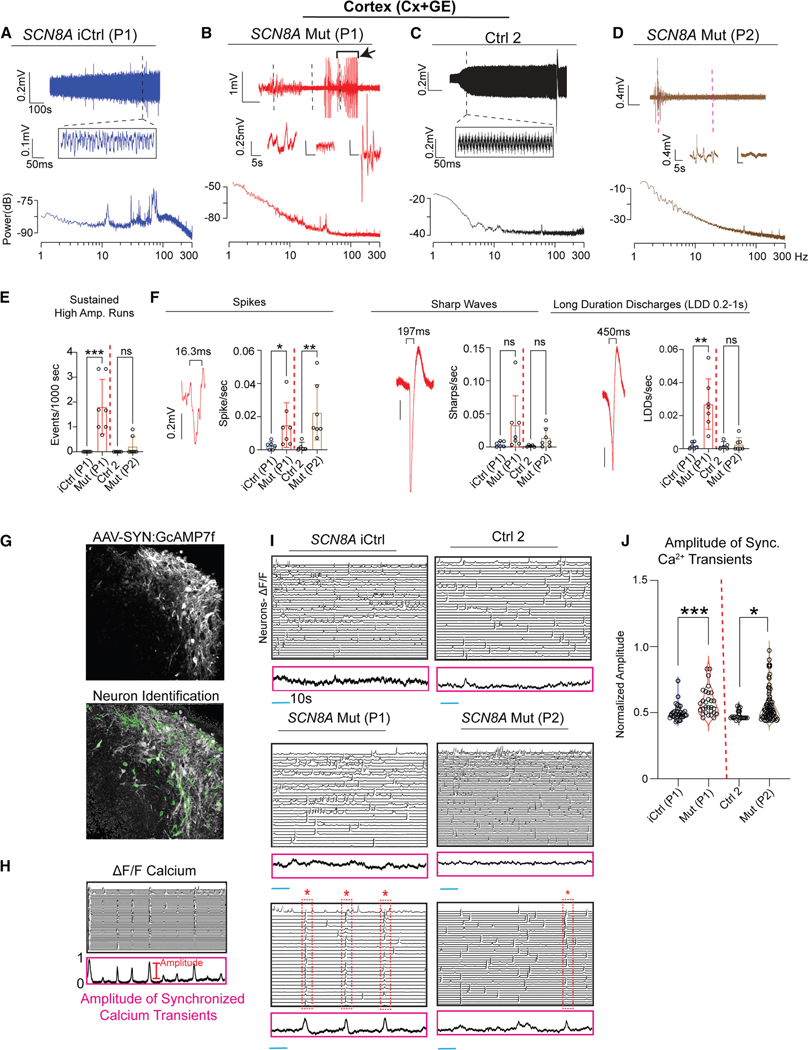
DEE-13 cortical assembloids demonstrate network hyperexcitability (A) Raw trace (top) and time expansion (middle) from an LFP recording of an isogenic control (iCtrl, P1) p.SCN8A cortex assembloid (Cx+GE). Bottom: periodogram from the entire raw trace. (B) Raw trace (top) and three time expansions (middle) from the LFP recording of a Mut P1 (p.R1872>L) p.SCN8A Cx+GE assembloid, with time expansions near the dotted lines in the raw trace. Bottom: periodogram from the entire raw trace. (C) Raw trace (top) and time expansion (middle) from an LFP recording of a control (Ctrl 2) cortex assembloid (Cx+GE). Bottom: periodogram from the entire raw trace. (D) Raw trace (top) and two time expansions (middle) from the LFP recording of a Mut P2 (p.V1592>L) p.SCN8A Cx+GE assembloid, with time expansions near the dotted lines in the raw trace. Bottom: periodogram from the entire raw trace. (E) Number of sustained high amplitude events/1000s in iCtrl, Ctrl 2, and Mut, defined as sustained runs ≥30 s with root mean square amplitude ≥5×greater than the preceding 30 s. Individual datapoints along with their mean ± SD are shown. ***p = 0.0004. (F) Frequency of spikes, sharp waves, and LDDs from iCtrl, Ctrl 2, and Mut Cx+GE LFP recordings, with a representative example of each discharge shown in red. For (E) and (F), *n* = 6 biologically independent samples (differentiations) for iCtrl, *n* = 5 for Ctrl 2, and *n* = 7 for Mut P1 and P2. Individual datapoints along with their mean ± SD are shown. *p = 0.0346, **p = 0.0071 (spikes), and **p = 0.0097 (LDDs); Kruskal-Wallis test. (G) Schematic for calcium transient identification and functional cluster generation after multiphoton imaging with a genetically encoded calcium indicator. (H) Normalized ΔF/F of calcium indicator activity in iCtrl Cx+GE. Each line represents a single neuron’s activity. (I) Representative normalized ΔF/F of calcium indicator activity in iCtrl, Ctrl 2, Mut P1, and Mut P2 Cx+GE. Controls (top) show decorrelated traces with minimal synchrony, whereas Mut Cx+GE has both decorrelated (top) and highly synchronous discharges (bottom). (J) Violin plots show a significant increase in average amplitude of Ca^2+^ transients in iCtrl vs. Mut P1 and Ctrl 2 vs. Mut P2 Cx+GE. Linear mixed effects model. ***p = 0.00024 (iCtrl vs. Mut P1) and *p = 0.0332 (Ctrl 2 vs. Mut P2); each dot represents a separate recording. *n* = 7 independent differentiations for iCtrl and Mut P1, and *n* = 5 independent differentiations for Ctrl 2 and Mut P2.

To assess circuit-specific effects of gain-of-function p.SCN8A mutations in DEE-13, we performed extracellular local field potential (LFP) recordings and two-photon calcium imaging (2PCI) of Cx+GE assembloids derived from two DEE-13 patients with distinct SCN8A variants (p.R1872L in P1 and p.V1592L in P2).^7^ P1 exhibited severe, treatment-resistant epilepsy, while P2 had a milder clinical profile with well-controlled seizures.^7^ For comparison, we used a CRISPR-corrected isogenic control (iCtrl) for P1. Since we did not have a matched isogenic control for P2, we compared P2 to an unrelated “control 2” (Ctrl 2). Ctrl 2 was derived from the previously validated iPSCs of an isogenic control for a Tau variant (R406W).^27^ As in previous work,^4^ control assembloids displayed oscillatory LFPs resembling *in vivo* cortical rhythms (Figures 2A–2C). In contrast, both Mut P1 and Mut P2 assembloids exhibited high-amplitude LFP bursts with quiescent periods (Figures 2B–2D), with significantly more sustained high-amplitude discharges in Mut P1 relative to iCtrl. Quantification revealed increased spike rates in both mutants compared to their respective controls, with additional increases in long-duration discharges (LDDs) specific to Mut P1 (Figure 2E), consistent with the differing clinical severities.

To measure single-cell activity, we infected Cx+GE assembloids with AAV1 Syn:GCaMP7f virus (Figure 2F) and recorded spontaneous calcium activity as changes in GCaMP7f fluorescence (ΔF/F) 2 weeks later. To quantify network-wide hyper-synchronization associated with an “epileptic-like” hyperexcitable state, we calculated the percentage of cells simultaneously active in each frame (Figure 2G). Network-wide activity was largely desynchronized in both iCtrl and Ctrl 2 Cx+GE assembloids (Figure 2H), whereas both Mut P1 and Mut P2 Cx+GE exhibited asynchronous activity interspersed with periods of network-wide synchronization (Figure 2H; Videos S1, S2, and S3). The amplitude of synchronized calcium transients (i.e., percentage of active cells per frame) was significantly increased in Mut P1 (relative to iCtrl) and Mut P2 (relative to Ctrl 2) Cx+GE (Figure 2I).

**Figure 3.**
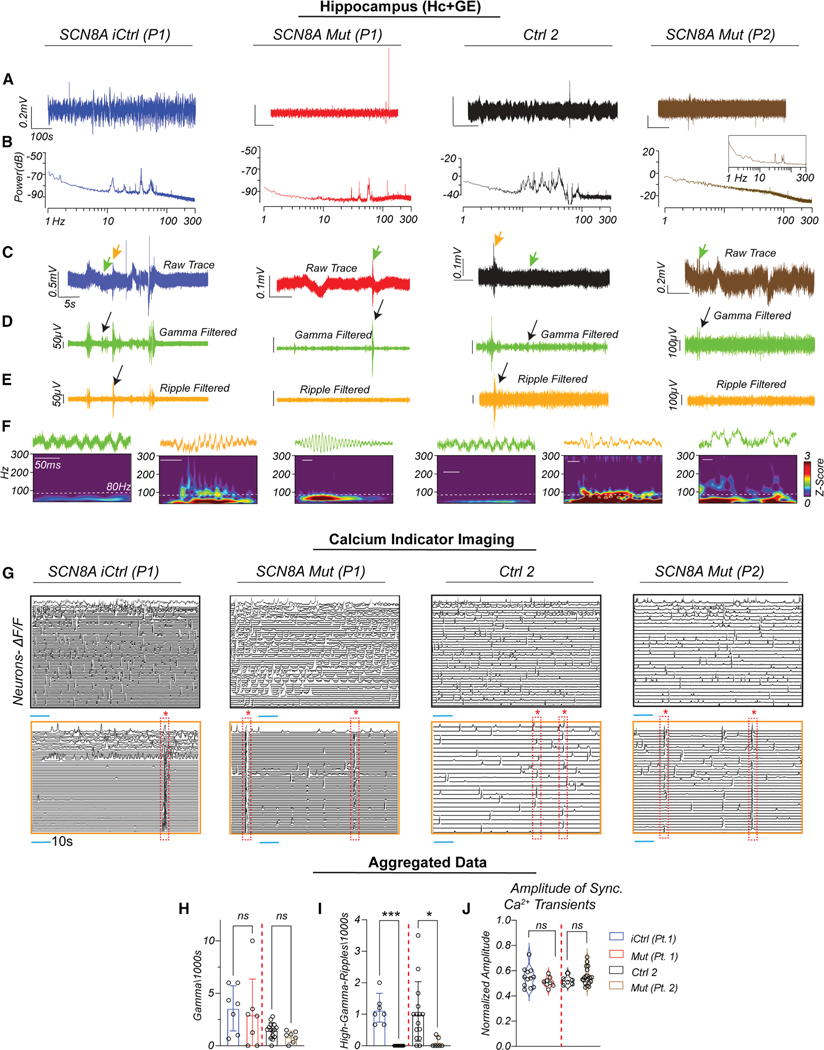
DEE-13 hippocampal assembloids show a reduction in fast oscillations but lack overt hyperexcitability (A) Raw traces from LFP recordings of iCtrl (P1, blue), Mut P1 (p.R1872>L, red), Mut P2 (p.V1592> L, brown) p.SCN8A, and Ctrl 2 (black) hippocampal (Hc+GE) assembloids. (B) Corresponding power spectra (periodograms) from recordings in (A). iCtrl, Ctrl 2, and Mut P1 exhibited sustained oscillations lasting ≥ 300 s, whereas Mut P2 also had oscillations but briefer (∼60 s) bouts of rhythmic activity (inset). (C) Time-expanded raw traces highlighting candidate oscillatory events. (D) Gamma-filtered (30–80 Hz) traces from segments in (C), with black arrows indicating putative gamma bursts. (E) Ripple-filtered (80–160 Hz) traces from (C), with high gamma/ripple events observed only in iCtrl and Ctrl 2 (black arrows). Mut P1 and P2 exhibited slower low gamma activity without discrete high gamma/ripple peaks. (F) Morlet wavelet spectrograms from events in (C)–(E), showing gamma and high gamma/ripple power over time. (G) Representative ΔF/F calcium imaging traces from iCtrl (P1), Mut P1, Ctrl 2, and Mut P2 Hc+GE assembloids. Top: decorrelated neuronal activity. Bottom: synchronized network events (red dashed boxes). (H) Quantification of gamma bursts (low gamma) per 1000 s showed no significant differences across groups. Mean ± SD are shown, with each dot representing an individual differentiation. A test for normality was followed by a Kruskal-Wallis test. (I) Quantification of high-gamma/ripple bursts per 1000 s revealed significantly more events in iCtrl compared to Mut P1 (***p = 0.0008) and in Ctrl 2 compared to Mut P2 (*p = 0.0170); mean ± SD are shown with each dot representing an individual differentiation. A test for normality was followed by a Kruskal-Wallis test. (J) Quantification of synchronized calcium transient amplitudes showed no significant change in network synchrony in Mut P1 compared to iCtrl or in Mut P2 compared to Ctrl 2 (linear mixed effects model). Each dot represents an individual recording, and the violin plot shows the distribution of the data; *n* = 6 independent differentiations for iCtrl, Mut P1, and Mut P2; and *n* = 5 for Ctrl 2.

Unlike Cx+GE assembloids, which showed clear evidence of hyperexcitability in the presence of gain-of-function p.SCN8A mutations, Hc+GE assembloids from the same patients did not exhibit overt epileptiform activity. LFP recordings from Hc+GE assembloids derived from both Mut P1 and Mut P2 lacked high-amplitude bursts or spikes, similar to both controls (Figure 3A). Power spectral analysis of iCtrl and Mut P1 Hc+GE assembloids showed sustained low-frequency oscillations persisting for at least 5 min, without consistent differences across frequency bands (Figures 3B and S2A). Mut P2 LFPs also displayed low-amplitude oscillatory activity, typically lasting about 1min per epoch (inset, Figure 3B). These findings suggest that the network-level hyperexcitability observed in Cx+GE assembloids is not uniformly present across brain regions and may depend on region-specific circuit composition.

Given the absence of clear hyperexcitability, we evaluated whether iCtrl Hc+GE assembloids generated canonical hippocampal circuit activities and whether these were altered in p.SCN8A variant-expressing assembloids. We first looked for sharp wave ripples (SWRs), high-frequency oscillations riding on a high-amplitude discharge, as these are well-established hippocampal activities associated with memory consolidation.^28,29^ We identified all discharges in raw LFP traces and filtered traces in the low gamma (30–80 Hz) (Figure 3D) and high gamma/ripple (80–160 Hz) (Figure 3E) ranges. Although gamma epochs were observed in all Hc+GE lines (Figure 3D), high gamma/ripple epochs were almost exclusively in both control Hc+GE lines (Figure 3E) and absent from Mut specimens. Morlet plots confirmed only low gamma frequency discharges in Mut P1 and P2, with additional higher frequency gamma-ripple in iCtrl and Ctrl 2 (Figure 3F). Notably, we did not find any evidence of activity resembling classic SWRs. Quantification showed no significant changes in low gamma activity (Figure 3G), but notable reductions in high gamma/ripple activity in Mut P1 compared to iCtrl and in P2 compared to Ctrl 2 (Figure 3H).

Similar to initial LFP results (Figures 3A and 3B) and unlike Cx+GE, calcium indicator data showed comparable synchronous and asynchronous activity across iCtrl, Mut P1, Ctrl2, and Mut P2 Hc+GE (Figure 3I; Videos S4, S5, and S6). Quantification revealed no significant differences in synchronized events (Figure 3J).

### Aberrant network activity in DEE-13 Hc+GE assembloids resembles disruptions seen in epilepsy patients

**Figure 4.**
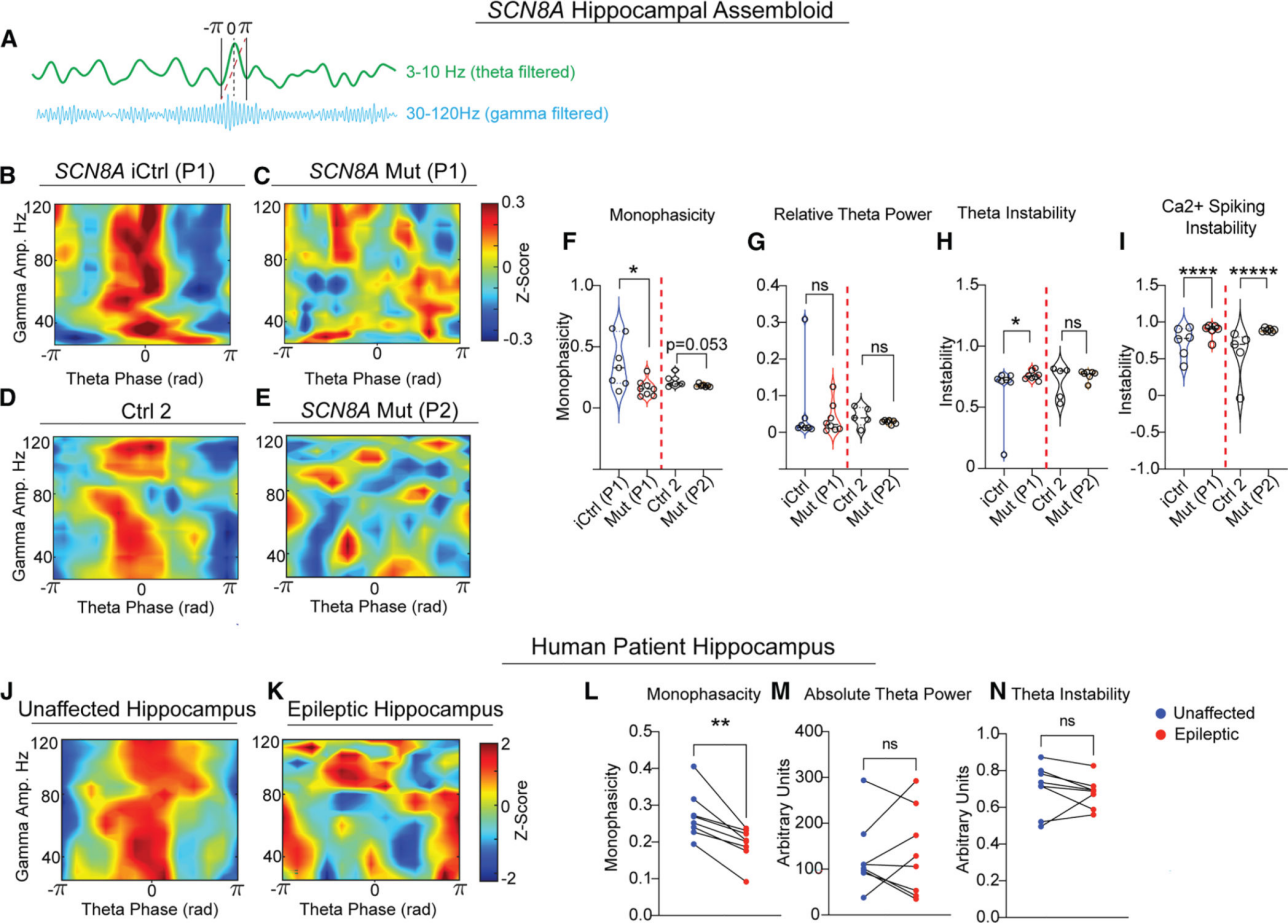
Perturbation of theta-gamma coupling in both DEE-13 hippocampal assembloids and human temporal lobe epilepsy (A) Example of phase-amplitude coupling (PAC) in an iCtrl Hc+GE assembloid showing higher gamma amplitude coupled to the 0 phase of theta (3–10 Hz). (B–E) Representative heatmaps of theta phase and gamma amplitude coupling in p.SCN8A P1 iCtrl (B), Ctrl 2 (D), and Mut P1 and P2 (C and E) Hc+GE assembloids. Both iCtrl and Ctrl 2 assembloids exhibit monophasic PAC, whereas both Mut assembloids exhibit disordered PAC. (F–I) Quantification of monophasicity (F), theta power (G), theta instability (H), and Ca^2+^ spiking instability (I) in iCtrl vs. Mut P1 and Ctrl 2 vs. P2 Mut Hc+GE assembloids. The monophasicity is significantly reduced in P1 relative to iCtrl (*p* = 0.0103) with a trending reduction in P2 relative to Ctrl 2 (*p* = 0.053). No significant differences in theta power were found. P1 Mut dynamics show increased instability relative to iCtrl both at the level of theta oscillations (*p = 0.0361) (H) and Ca^2+^ spiking (****p < 0.0001) (I), while P2 Mut only shows increased instability relative to Ctrl 2 at the level of Ca^2+^ spiking (****p < 0.0001) (I). Violin plots with datapoints (average for each assembloid) are shown in (F)–(H) (LFP data); n = 5–8 independent differentiations/genotype, one-tailed Wilcoxon rank-sum test. Violin plot with datapoints (average for each assembloid) is shown in I (two-photon imaging); n = 5–6 independent differentiations/genotype, linear mixed effects model. (J and K) PAC heatmaps in the healthy (I) and epileptic (J) hippocampus of a single patient. The healthy side shows highly monophasic coupling, whereas the epileptic hippocampus from the same patient shows disordered coupling. (L–N) Monophasicity (L), theta power (M), and theta instability (N) in healthy and epileptic hippocampi of eight temporal lobe epilepsy patients. Monophasicity was reduced in the epileptic hippocampi of each of the eight patients. No differences were noted in theta power/instability. One-tailed Wilcoxon signed rank paired t test, *n* = 8 patients, **p = 0.0039.

Theta-gamma phase-amplitude coupling (PAC) is crucial in the mammalian hippocampus for encoding, maintenance, and retrieval of information.^30^ To further explore network dysfunction in Mut Hc+GE assembloids, we analyzed LFPs for coupling between hippocampal theta (3–10 Hz) phase and gamma/high gamma (30–120 Hz) amplitude (Figure 4A). No difference in theta-gamma coupling strength was observed between iCtrl and Mut 1 or between Ctrl 2 and Mut 2 Hc+GE assembloids (Figure S2B). However, the coupling pattern was more “monophasic” in both iCtrl (Figure 4B) and Ctrl 2 (Figure 4D), meaning the entire range of gamma amplitudes tended to lock to the same theta phase, typically the peak. In contrast, this phase relationship was disordered in both P1 and P2 Mut Hc+GE (Figures 4C–4E), with significant or nearly significant reduced monophasicity (Figure 4F), despite no difference in theta power (Figure 4G) or consistent change in gamma power (Figure S2A) across all p.SCN8A lines. Stability analysis using the K-statistic from the 0–1 chaos test^31,32^ showed theta oscillations were significantly less stable in Mut Hc+GE compared to iCtrls (Figure 4H), with no difference between Ctrl 2 and Mut 2 Hc +GE. Unstable single-cell spiking patterns derived from 2PCI were also observed in both Mut 1 relative to iCtrl and in Mut 2 relative to Ctrl 2 Hc+GE (Figure 4I).

To assess the *in vivo* relevance of the observed PAC deficits, we analyzed theta-gamma coupling in bilateral intracranially recorded LFPs from the hippocampi of eight mesial temporal lobe epilepsy (mTLE) patients during task-free and seizure-free periods. Remarkably, reduced monophasicity in theta-gamma coupling was found in each patient’s epileptic hippocampus compared to their healthy hippocampus (Figures 4J–4L). These results suggest that loss of monophasic theta-gamma coupling, first observed in our Hc+GE assembloids, may be a hallmark of hippocampal circuit dysfunction in epilepsy. Like our Mut Hc+GE assembloids, there was no change in theta power in the patients’ epileptic hippocampi (Figure 4M). However, unlike our P1 Mut vs. iCtrl assembloids, there was no clear increase in theta oscillation instability (Figure 4N).

### *In silico* modeling predicts changes in cell numbers

**Figure 5.**
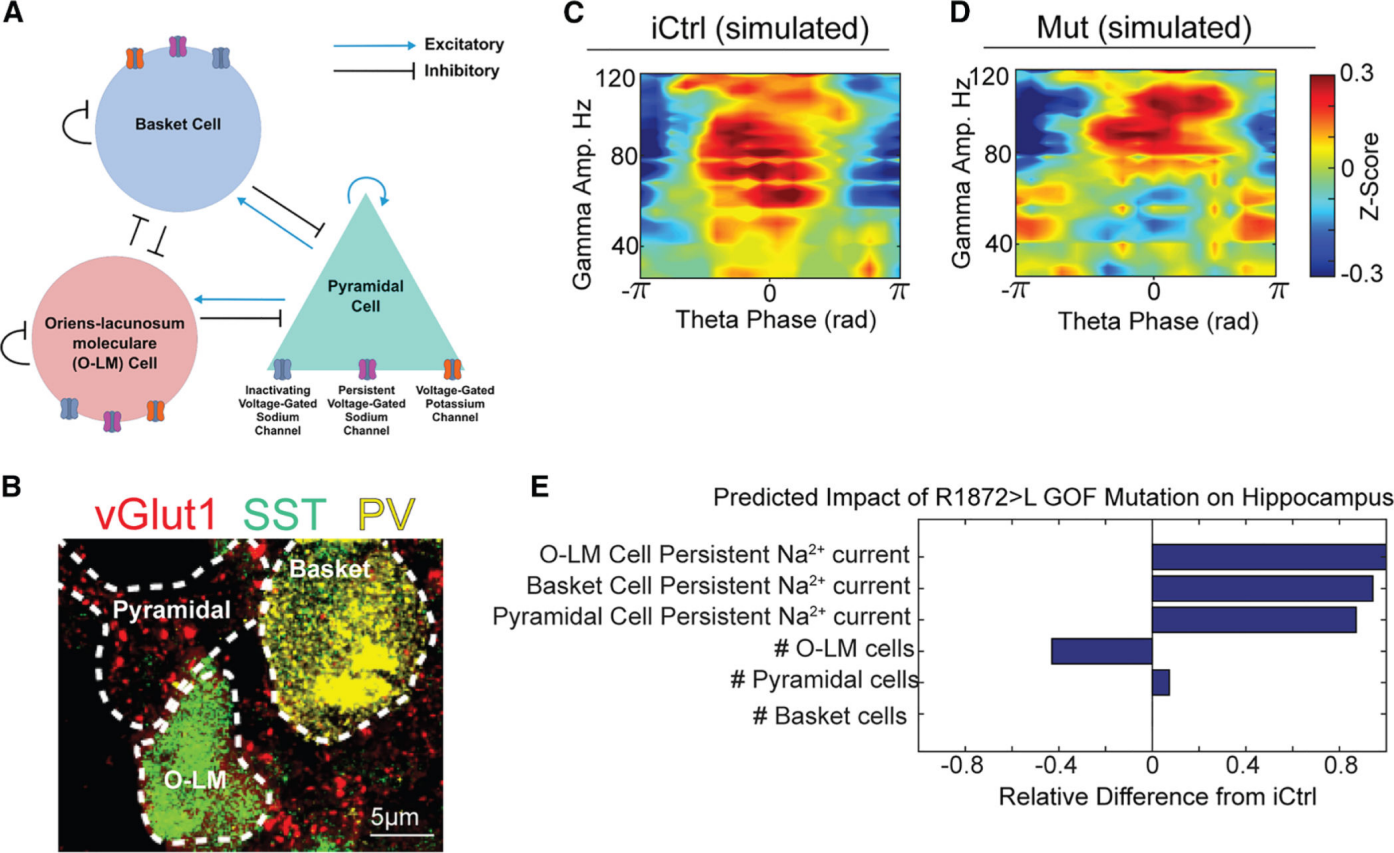
In silico model predicts loss of O-LM cells (A) Schematic of the CA3 microcircuit model, incorporating pyramidal cells, parvalbumin-expressing basket cells, and somatostatin-expressing O-LM interneurons. Synaptic connectivity and cell-intrinsic conductance were modeled using Hodgkin-Huxley equations (see STAR Methods). (B) IHC of Hc+GE assembloids shows spatial proximity of vGlut1^+^ pyramidal neurons/excitatory pre-synpatic puncta (red), SST^+^ O-LM cells (green), and PV^+^ basket cells (yellow), supporting the plausibility of local circuit/synaptic interactions between the modeled populations. Additional examples with a full field of view are in Figure S1F; these tripartite ensembles were observed in 41/46 regions of interest from four imaged sections. Scale bars, 5 μm. (C and D) Phase-amplitude coupling plots from iCtrl and Mut simulations show theta-gamma interactions consistent with the *in vitro* LFP recordings. (E) Predicted impact of the p.SCN8A R1872>L gain-of-function mutation on the hippocampal circuit parameters, including cell-intrinsic persistent sodium currents and population-level changes. The model predicts a substantial loss of O-LM cells, increased pyramidal cell number, and increased persistent Na^+^ current across all modeled cell types.

To predict the mechanisms underlying the electrophysiological phenotype of Mut Hc+GE assembloids, we developed an *in silico* CA3 circuit simulation of DEE-13 by extending a previous model^33^ to include persistent sodium currents and incorporating fixed parameters like cell numbers based on preliminary immunohistochemistry (IHC) data. This model included fast-spiking basket cells, slow-spiking oriens-lacunosum/moleculare (O-LM) cells, and pyramidal cells, each described by Hodgkin-Huxley equations (see STAR Methods) (Figure 5A). Critically, while we cannot directly confirm circuit connectivity among these cell types, IHC staining for parvalbumin (PV) (basket cells), somatostatin-expressing (SST) (O-LM cells), and vesicular glutamate transporter 1 (vGlut1, excitatory puncta on pyramidal neurons) revealed that these populations are spatially adjacent within the Hc+GE assembloids (Figure 5B), supporting the plausibility of synaptic interactions between them. We then utilized machine learning (namely, a genetic optimization algorithm) to tune the model to recreate the electrophysiological phenotypes observed in our control and DEE-13 Hc+GE assembloids (Figures 5C, 5D, and S3). The resulting Mut model exhibited significant increases in persistent sodium currents across all cell types (a known effect of gain-of-function p. SCN8A mutations^7,34–38^), loss of O-LM cells, and an increase in pyramidal cells, with no changes in basket cells (Figure 5E).

Critically, when the normal persistent sodium current was restored in our model, monophasic theta-gamma coupling only partially recovered (Figure S3A), indicating that normal cellular composition (like in iCtrl) is crucial for monophasic theta-gamma coupling in DEE-13 Hc+GE assembloids. Notably, the dysfunction of SST hippocampal interneurons, including O-LM cells, disrupts circuitry and impairs memory in disorders like Rett syndrome,^39^ and O-LM cell loss has been noted in multiple TLE models.^40–42^ However, the cellular composition of the hippocampus in DEE-13 has not been previously evaluated.

### p.SCN8A Hc+GE assembloids display alterations in interneuron and excitatory neuron development

**Figure 6.**
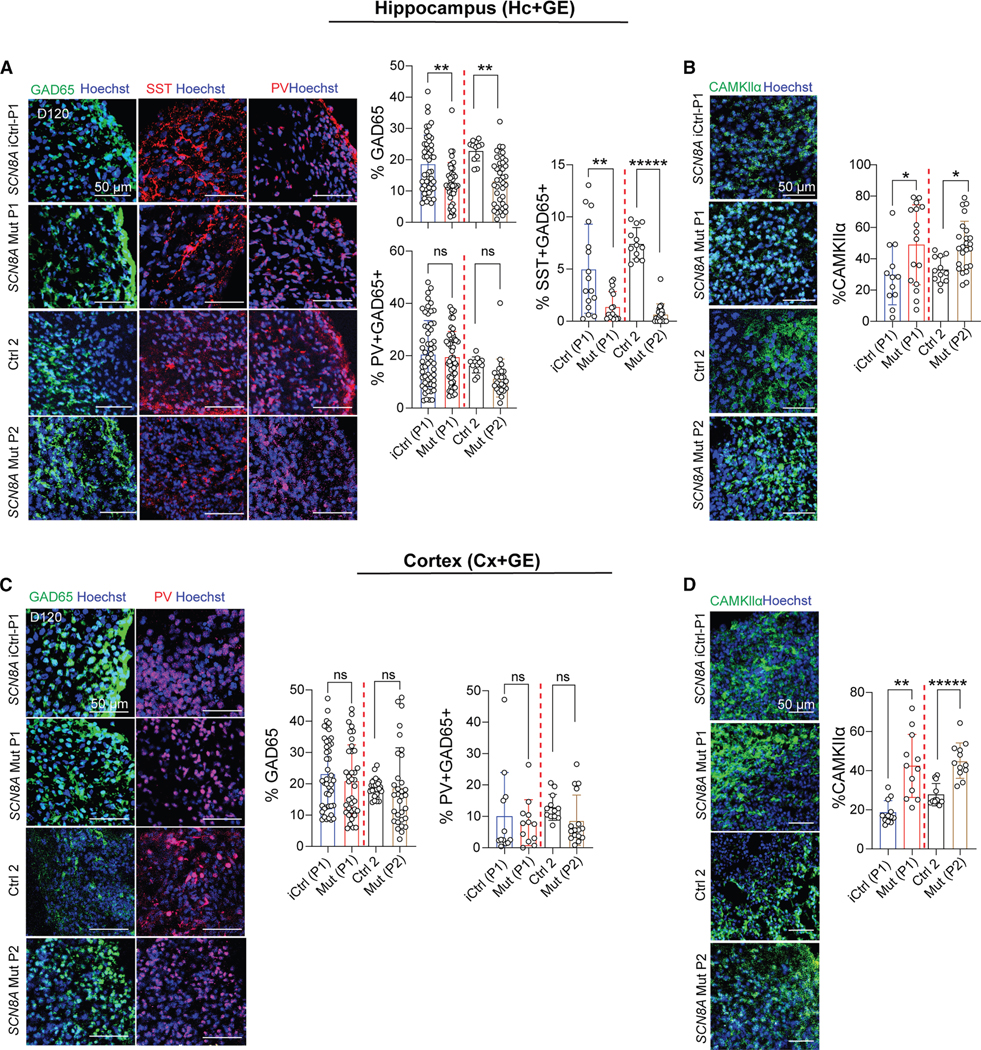
Hippocampal but not cortical DEE-13 assembloids demonstrate reduced inhibitory interneuron numbers (A) IHC analysis of Hc+GE assembloids reveals a reduction in the pan-inhibitory interneuron marker glutamic acid decarboxylase-65 (GAD65/GAD-2) and in the somatostatin (SST) subtype of interneurons in both Mut P1 and Mut P2 compared to iCtrl or Ctrl 2, respectively. No significant reductions were observed in parvalbumin+ (PV+) interneurons. (B) IHC analysis for excitatory neurons with CAMKII-*α* revealed a significant increase in both Mut P1 and Mut P2 compared to iCtrl or Ctrl 2, respectively. *n* = 3–14 independent differentiations per genotype and four sections per assembloid with ≥ 4,280 cells per sample. For Mut P1 and Mut P2 respectively; GAD65 **p = 0.0031 **p = 0.00124; SST **p = 0.0025 and *****p = 2.40E-16; CAMKII-*α* *p = 0.039 and *p = 0.0149. (C) IHC for both GAD65 and PV in Cx+GE assembloids reveals no significant differences between iCtrl vs. Mut P1 or Ctrl 2 vs. Mut P2. (D) IHC for CAMKII-*α* reveals a significant increase in both Mut P1 and Mut P2 compared to iCtrl or Ctrl 2, respectively. *n* = 3–11 independently generated assembloids per genotype, four sections per assembloid with ≥ 2,681 cells per sample. For Mut P1 and Mut P2, respectively, CAMKII-*α* **p = 0.0028 and *****p = 6.27E-6. For (A–D), all datapoints with their mean ± SD are shown; significance calculations used a linear mixed effects model; and Scale bars, 50 μm.

Since our computational model predicted that the aberrant electrophysiology in p.SCN8A Mut Hc+GE assembloids may be driven by changes in the balance of inhibitory O-LM and excitatory pyramidal cells, we employed IHC and snRNAseq approaches to evaluate the cellular composition of p.SCN8A iCtrl versus Mut cortical and hippocampal assembloids, focusing on the *in vitro* equivalent of *in silico* “basket” and O-LM interneurons alongside excitatory neurons (ExNs). We quantified the expression of GAD65, PV (fast-spiking/basket interneurons), and SST (O-LMs express SST), along with calcium/calmodulin-dependent protein kinase II subunit α (CAMKII-*α*, encoded by CAMK2A) to distinguish the ExNs. Mut P1 and P2 Hc+GE exhibited a significant reduction in SST+ but not PV+ interneurons, and a significant increase in CAMKII-*α* + ExNs compared to their respective controls (Figures 6A and 6B). The changes in these cell proportions are consistent with our computational model’s predictions regarding the cellular causes of reduced theta-gamma monophasicity (Figure 5E). IHC for Cx +GE showed no reduction in total (GAD65+) interneurons (Figure 6C). In case this result masked an interneuron subtype change, we also examined PV and noted no significant changes (Figure 6C). However, a significant increase in CAMKII-*α* + ExNs was noted in both P1 and P2 Mut Cx+GE compared to iCtrl or Ctrl 2 Cx+GE, respectively (Figure 6D).

### Transcriptomic analysis reveals selective Hc+GE interneuronopathy in p.SCN8A Mut Hc+GE assembloids

**Figure 7.**
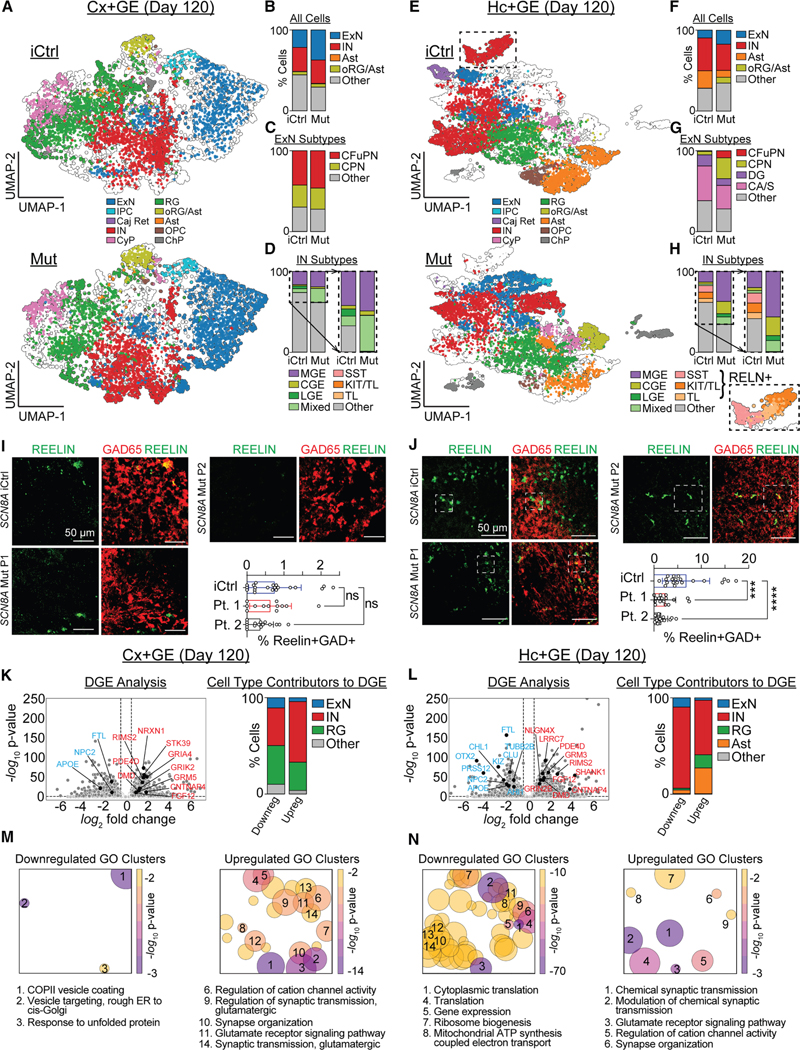
DEE-13 differentially affects circuit composition and gene expression in day 120 cortical versus hippocampal assembloids (A) 2D UMAP of cells from day 120 iCtrl and Mut P1 Cx+GE assembloids revealed excitatory neurons (ExN), intermediate progenitors (IPCs), Cajal-Retzius (Caj Ret), inhibitory neurons (INs), cycling progenitors (CyPs), radial glia (RG), outer radial glia (oRG), astrocytes (Asts), oligodendrocyte precursors (OPCs), and choroid plexus (ChP). (B) The Mut P1 Cx+GE assembloids show more ExNs compared to iCtrl. “Other” cell types (IPC, Caj Ret, CyP, RG, OPC, and ChP) are grouped for clarity. (C) No significant changes in the corticofugal (CFuPN) or callosal (CPN) projection ExN subtypes between Mut P1 and iCtrl Cx+GE. “Other” excitatory neuron subtypes include immature neurons (see Figure S5A). (D) Mut P1 Cx+GE assembloids show more mixed CGE- and LGE-like INs compared to iCtrl. Note that medial ganglionic eminence-like (MGE) INs include MGE types 1–4 described in Figure S5B. “Other” inhibitory interneuron subtypes include all immature and unspecified interneurons (see Figure S5B). (E) UMAP of cells from day 120 iCtrl and Mut P1 Hc+GE assembloids. (F) Mut P1 Hc+GE assembloids show more ExNs, fewer INs, fewer Asts, and more co-clustered oRG/Asts compared to iCtrl. (G) Mut P1 Hc+GE assembloids show more cortex-like CFuPN and CPN ExNs and fewer hippocampal dentate granule-like (DG) and cornu ammonis/subiculum-like (CA/S) ExNs compared to iCtrl. (H) Mut P1 Hc+GE assembloids lack *RELN*+ INs present in iCtrl Hc+GE. (I and J) IHC shows few cells co-expressing Reelin and GAD65 in cortical assembloids across iCtrl, Mut P1, and Mut P2 lines, whereas hippocampal assembloids show a substantial population in iCtrl that is lost in Mut lines (datapoints are shown with their mean ± SD; Mut P1 ***p = 0.000237, Mut P2 ****p < 0.0001, linear mixed effects model, five iCtrl, four Mut P1 and P2 assembloids, four sections per assembloid, and ≥ 6,324 cells/sample). Scale bars, 50 μm. (K and L) Differential gene analysis volcano plots of day 120 Cx+GE and Hc+GE, respectively. Selected upregulated genes (red) in Mut are involved in glutamatergic pathways and likely promote a hyperexcitable state. Reduced expression of selected downregulated genes (blue) has been associated with epilepsy and intellectual disability. The upregulated and downregulated genes are shown in relative proportions. Gene expression data are provided in Table S3. (M and N) Gene ontology (GO) analysis of Cx+GE and Hc+GE, respectively, shows upregulation of glutamatergic pathways and downregulation of basic cellular processes in the hippocampal Mut P1 assembloids. Circle sizes correspond to the number of GO terms. GO data are provided in Table S4.

To further explore the divergent developmental trajectories between iCtrl and Mut Cx+GE and Hc+GE assembloids, we focused on P1 (R1872>L p.SCN8A variant), utilizing an expanded snRNAseq dataset that included both iCtrl and Mut P1 Cx+GE and Hc+GE assembloids at day 120 and subsequently added iCtrl and Mut P1 Hc+GE at day 84. Cx+GE assembloids comprised diverse but expected cell types (Figure 7A compared to Figures S4A, S4B, and S5). Consistent with IHC results (Figures 6C and 6D), Mut Cx+GE assembloids showed a relative increase in ExNs (Figure 7B) without significant changes in other major cell populations, including INs (Figure 7B). No substantial changes were observed in the ExN subtypes between iCtrl and Mut (Figure 7C), but there was an increase in mixed caudal ganglionic eminence-/lateral ganglionic eminence-like (CGE/LGE-like) inhibitory interneurons (INs) in Mut assembloids (Figure 7D). Hc+GE assembloids also comprised diverse cell types (Figure 7E), but demonstrated substantial differences between Mut and iCtrl. Mut Hc+GE showed a relative increase in ExNs (Figure 7F), consistent with IHC and *in silico* modeling predictions. Notably, iCtrl Hc+GE exhibited a well-defined astrocyte population (i.e., *GFAP*+, *AQP4*+, and *S100B*+) that decreased in Mut Hc+GE alongside an increase in the mixed outer radial glia/astrocyte co-cluster abundant in Cx+GE (Ast and oRG/Ast, Figure 7F). Cajal-Retzius cells were also largely exclusive to iCtrl Hc+GE. Subtype analysis of excitatory clusters indicated that changes in Mut Hc+GE were driven by increased proportions of excitatory cells expressing cortical-like deep and upper layer markers (CFuPN and CPN), with a relative reduction in ExNs expressing hippocampal DG-like and CA/S-like markers (Figure 7G). These findings suggest that the gain-of-function p.SCN8A mutation may drive hippocampal cell identities toward a cortical-like fate.

The iCtrl Hc+GE assembloids contained a unique *RELN*-expressing interneuron cluster (boxed cluster, Figure 7E) that was absent in Mut Hc+GE (and all Cx+GE). Consequently, Mut Hc+GE exhibited a substantial overall reduction in inhibitory INs (Figure 7F), unlike Cx+GE. Subtype analysis indicated that the *RELN*-expressing interneuron cluster, selectively reduced in Mut Hc+GE, included *SST* interneurons (and thus putative O-LMs) and *CHRM2*-expressing putative trilaminar (TL) interneurons (Figure 7H). The reduction in (*RELN*+) SST interneurons aligns with our *in silico* predictions (Figure 5E) and IHC results (Figures 6A and 6B). TL interneurons are closely associated with ripple activity,^43^ so their reduction may explain the observed decrease in high gamma/ripple oscillations in Mut Hc+GE (Figure 3). Subsequently, IHC confirmed limited Reelin+ interneurons in Cx+GE (Figures 7I and S1E) and showed that this population was present in iCtrl Hc+GE but significantly reduced in both P1 and P2 Mut Hc+GE (Figures 7J and S1E). Notably, snRNAseq analysis from both iCtrl and Mut day 84 Hc+GE demonstrated the absence of this *RELN*-expressing cluster (Figure S6), suggesting that it is a later-developing IN population in iCtrl Hc+GE that likely fails to develop completely in Mut Hc+GE.

To explore the mechanisms underlying divergent cellular development due to a *p.SCN8A* variant in the hippocampus vs. cortex-like assembloids, we performed differential gene expression (DGE) and gene ontology (GO) analysis. DGE analysis of upregulated genes revealed an increase in synapse-related genes in both Cx+GE (Figure 7K) and Hc+GE (Figure 7L), suggesting a potential mechanism for hyperexcitability in DEE-13. Notably, INs rather than ExNs were the primary contributors to the genes upregulated in Cx+GE and Hc+GE (Figures 7K and 7L). This implies some degree of interneuron dysfunction and a possible shift away from the excitatory-inhibitory balance in both brain regions. In contrast, DGE analysis of downregulated genes in Mut Cx+GE and Hc+GE demonstrated regional-specific changes. For example, INs were the primary contributors to the genes downregulated in Hc+GE but not Cx+GE (where many cell types contributed). Moreover, Mut vs. iCtrl Hc+GE showed a larger depletion of genes important for normal development compared to Mut vs. iCtrl Cx+GE, and many of these genes are associated with epilepsy or intellectual disability^44^ when dysfunctional (Figures 7K and 7L). Interestingly, inhibitory neuron co-expression network analysis (hdWGCNA)^45^ revealed one module overlapping with many of these same genes that were localized to *RELN*+ interneurons (Figure S7). This suggests that the region-specific gene changes in Mut Hc+GE may be largely related to changes in *RELN*+ interneurons. GO analysis confirmed upregulation of synapse-related genes in both Mut Cx+GE and Hc+GE assembloids and further revealed that these genes were primarily linked to excitatory synaptic function (Figures 7M and 7N). Additionally, GO analysis revealed few downregulated pathways in Mut vs. iCtrl Cx+GE (Figure 7M) but many in Mut vs. iCtrl Hc+GE (Figure 7N) that included critical cellular viability/housekeeping tasks such as translation, gene expression, metabolism, etc. Together, these findings indicate that p.SCN8A leads to two distinct changes, with interneurons as primary contributors: (1) nonspecific upregulation of hyperexcitability-associated genes, which may contribute to excitatory-inhibitory imbalance, and (2) hippocampal-specific downregulation of essential genes that are involved in critical cell functions and that may contribute to phenotypic developmental abnormalities when they are dysfunctional. Lastly, we considered the possibility of a pure cell-autonomous effect due to differential expression of p.SCN8A between iCtrl and Mut assembloids; however, p.SCN8A expression did not substantially differ between day 120 iCtrl and Mut Cx+GE nor between day 120 iCtrl and Mut Hc+GE, even within the ExN and IN populations only (Figure S4C).

## DISCUSSION

Collectively, our studies point to significant region-specific differences and the impact of p.SCN8A variants on the developing brain. Whereas Mut Cx+GE assembloids exhibited hyperexcitability, Mut Hc+GE assembloids displayed a loss of monophasic theta-gamma coupling, a pattern also seen with *in vivo* intracranial depth recordings in healthy versus epileptic human hippocampi. These findings substantially expand our understanding of DEE-13 beyond mouse models, which have primarily focused on the seizure activities attributed to the dysfunction of ExNs in the forebrain, without investigation of hippocampal dynamics.^36,46^ The aberrant phase coupling seen in the assembloid models underscores the potential of these SCN8A variants to disrupt hippocampal function and suggests that hippocampal-dependent cognitive deficits may occur without cortical seizure-induced pathology. This is consistent with observations in a number of genetic epilepsies, including SCN8A, that cognitive deficits precede seizure onset or occur in the absence of significant seizure burden.^2,3^

Our experiments also reveal a selective vulnerability of putative O-LM and TL interneurons in Mut Hc+GE and a global increase and change in identity in ExNs in Mut assembloids. Although this was predicted by our *in silico* computational model and is consistent with the electrophysiological data, this finding is nevertheless unexpected based on prior animal studies where targeted expression of p.SCN8A in inhibitory interneurons was non-pathologic.^47^ The connection between alterations in sodium channel function and cell fate choices is not without precedent, as prior studies have uncovered neurogenic defects associated with pathogenic SCN3A variants^48^ and alterations in neural progenitor activities upon manipulation of membrane potentials.^49^ From a therapeutic standpoint, this indicates not only the importance of regionally targeted treatment, but also that normalizing channel function with traditional anti-seizure medications or with newer ion channel targeting anti-sense oligonucleotides^50^ may be insufficient to overcome pathogenic channel-driven cell fate changes and achieve complete disease remission. The only exceptions would be the most extreme cases, where full normalization of the channel is achieved and maintained at the earliest stages of development. In many cases, this would be *in utero* and too late for patients living with these disorders.

With that in mind, a strategy of interneuron replacement might be warranted given its demonstrated efficacy in animal models.^51^ However, even this approach could have limitations given our findings that excitatory-inhibitory neuron interactions can shape the composition of interneuron populations. Expression differences in excitatory genes may also play a role in establishing these region- and condition-specific developmental fates, as we observed a relative reduction in CA/DG excitatory markers and a relative increase in cortical excitatory markers (Figure 7G) in Mut Hc+GE compared to iCtrl. A recent study^52^ has shown that alterations in ExNs can induce interneurons to change their relative abundance and identity, including the expression of O-LM-associated markers. The specific excitatory populations that drive these interneuron changes merit further exploration in our model, as their identification could inform new therapeutic strategies with more enduring effects on interneuron/cell replacement approaches.

### Limitations of the study

While our simulations and *in vitro* experiments have provided valuable insights, this approach has limitations. The assembloids studied here model the developing cortex and hippocampus in isolation and lack the whole-brain interactions that occur *in vivo*. These interactions may play a role in both normal development and pathology. While our approach allowed us to more easily isolate region-specific pathologies, our findings would benefit from complementary whole-animal studies. It is well established that organoids, including our assembloids, represent early developmental stages. Even though we identify emergent network properties, these *in vitro* preparations likely do not capture the advanced neuronal maturation and synaptic complexity of *in vivo* circuits. This can be addressed with animal models where further neuronal maturity can be reached, and when possible, with human samples, such as postmortem tissue and human clinical electrophysiological recordings. The SCN8A variants were all derived from female donors. While we are not aware of a systematic impact of sex on DEE-13 phenotype, the use of female-derived iPSCs may restrict the generalizability of our findings. Moreover, our hippocampal simulation included only three cell types—O-LM cells, basket cells, and pyramidal cells—providing a simplified view that misses the full cellular diversity of the hippocampus. Although the model predicted O-LM cell loss, validated by IHC and snRNA-seq, the limited cellular diversity of the model warrants further *in vitro* exploration of cellular changes (for example, the TL interneurons that we identified in our snRNA-seq dataset and non-neuronal cells, such as astrocytes). Finally, the human patient data used for comparison were from individuals with adult-onset epilepsy, and not DEE-13, potentially leading to differences in network dynamics. This may explain why we did not observe a loss in theta stability in the human data, as we did in our Mut P1 hippocampal assembloids.

## RESOURCE AVAILABILITY

### Lead contact

Requests for further information and resources should be directed to and will be fulfilled by the lead contact, Ranmal A. Samarasinghe (rsamarasinghe@mednet.ucla.edu).

### Materials availability

This study did not generate new materials.

### Data and code availability

- All code used in data analysis was previously generated and published by others. Any minor modifications will be made publicly available at https://github.com/samarasinghelab at the time of publication.
- Raw and processed snRNAseq data generated in this study have been deposited in the NCBI Gene Expression Omnibus: https://www.ncbi.nlm.nih.gov/geo/query/acc.cgi?acc=GSE281622.
- Any additional information required to reanalyze the data reported in this paper is available from the lead contact upon request.

## STAR★METHODS

### EXPERIMENTAL MODEL AND STUDY PARTICIPANT DETAILS

#### hiPSC cultures

Human induced pluripotent stem cells (hiPSCs), reprogrammed from fibroblasts from two de-identified female DEE13 patients, were generated by the Parent lab at the University of Michigan as previously described.^7^ These hiPSCs were subsequently cultured using protocols employing pCXLE-hOCT3/4-shp53RNA, pCXLE-hUL, and pCXLE-hSK. The iCtrl for P1 was also generated and validated by Dr. Parent. Control 2 (Ctrl 2) is a p.R406W isogenic control (MAPT^+/+^) maintained by the Tau Consortium^57^ and was previously characterized and generously donated by Dr. Celeste Karch at the University of Washington SOM.^58^ The hiPSCs were derived from a de-identified patient (F11362.1) and reprogrammed from fibroblasts using a non-integrating Sendai virus carrying OCT3/4, SOX2, KLF4, and cMYC. The CRISPR-corrected isogenic control (used here) was generated at the same time. Quality control (QC) testing for the presence of the mutation by qPCR, chromosomal stability testing by G-band karyotyping, mycoplasma testing, and testing for the capacity to generate all three germ layers were performed as part of the Tau Consortium QC process for all hiPSCs and as detailed in.^57^ The identity of all lines was confirmed prior to shipment. All hiPSC experiments received prior approval from the University of California, Los Angeles (UCLA) Embryonic Stem Cell Research Oversight (ESCRO) committee and the Institutional Review Board.

#### Human patient local field potential recordings

We reanalyzed previously published data^59^ on hippocampal local field potentials from eight patients (four female, four male, ages 31–50, Table S1) who underwent stereotactic implantation of bilateral intracranial depth electrodes (Integra or Ad-Tech, 5 mm spacing) at the University of California, Irvine Medical Center to identify seizure onset zones for potential surgical intervention. Further demographic information for these subjects was not available. The study was approved by the institutional review boards at the University of California, Berkeley, and Irvine, with all subjects providing written informed consent. Electrode placements were based purely on clinical needs with MRI verification in the hippocampus. Electrode locations were confirmed using co-registered pre- and post-implantation T1-weighted MRI scans, with a high-resolution anatomical template (0.55 mm) aligned to each individual’s scan for precise localization. Only hippocampal recordings, specifically from DG/CA3 and CA1, were analyzed. Data were acquired with a Nihon Kohden system, filtered above 0.01 Hz, sampled at 5000 Hz, and analyzed using MATLAB with open-source toolboxes and custom scripts. During the initial study, participants viewed silent movie clips; however, here, we focused on task-free and seizure-free baseline hippocampal field potentials, examining theta-gamma phase-amplitude coupling monophasicity in each patient’s healthy and epileptic hippocampus.

### METHOD DETAILS

#### Organoid generation

Organoids representing the cortex (Cx), hippocampus (Hc), and ganglionic eminence (GE) were developed from the *p.SCN8A* mutant lines containing the R1872 > L (P1) or V1592 > L (P2) mutation, the CRISPR-corrected isogenic control for R1872 > L, or a previously published and validated CRISPR-corrected isogenic control for the MAPT Tau variant R406W. The latter was generously provided by the Tau Consortium/Dr. Celeste Karch. Organoids were induced toward a hippocampal fate by applying BMP4 along with CHIR 99021, a GSK3 inhibitor that acts as a Wnt agonist.^60^ A GE (ganglionic eminence) fate was achieved by using agonists of the Sonic hedgehog (Shh) pathway,^61^ while a cortical fate was established by omitting these specific patterning factors.^61^ Assembloids were created by fusing organoids of different types (Hc+GE and Cx+GE) using a modified version of the fusion protocol described in.^4^ On day 56, organoids were bisected and combined in microcentrifuge tubes containing 400 *μ* l of medium—N2B27 for Cx+GE and Neurobasal for Hc+GE. These tubes were then incubated at 37 ^◦^ C in a hyperoxic environment containing 5% CO_2_ and 40% O_2_ for 72 h. After incubation, fused assembloids were transferred to 24-well oxygen-permeable dishes (Lumox, Sarstedt) and maintained under similar conditions with media changes every other day. Neuronal migration experiments involved the infection of individual organoids with 5 *μ* l of approximately 1:98 × 10^13^ ml^− 1^ pAAV-CAG-tdTomato virus (Addgene, 59462-AAV1) on day 56. Fusion of the infected organoids was executed three days post-infection, as previously described.^4^ All experimental replicates represent assembloids derived from independent iPSC differentiations (referred to as “independently generated” above).

#### Immunohistochemistry

Assembloids were immersion fixed in 4% paraformaldehyde, cryoprotected in 30% sucrose, frozen in Tissue-Tek Optimal Cutting Temperature medium (Sakura) and cryosectioned. Immunostaining was performed using previously published laboratory protocols.^61^ Primary antibody staining was conducting using the following: rabbit anti-CAMK2 (ProteinTech, 13730–1-AP), 1:5,000 dilution; rat anti-CTIP2 (BCL11B; Abcam, ab18465), 1:1,000 dilution; rabbit anti-DLX1 (generous gift of S. K. Lee and J. Lee^62^), 1:3,000 dilution; guinea pig anti-DLX2 (generous gift of K. Yoshikawa and H. Shinagawa^63^), 1:3,000 dilution; mouse anti-GAD65 (BD Biosciences, 559931), 1:200 dilution; rabbit anti-GRIK4 (Invitrogen, PA5–111717), 1:100 dilution; mouse anti-NKX2–1 (Novocastra NCL-L-TTR-1), 1:500 dilution; goat anti-NRP2 (R&D Systems, AF2215), 1:40 dilution; rabbit anti-OLIG2 (EMD Millipore, AB9610), 1:5,000 dilution; rabbit anti-PAX6 (MBL International, PD022), 1:1,000 dilution; mouse anti-PROX1 (EMD Millipore, MAB5654), 1:200 dilution; rabbit anti-PV (Abcam, ab11427), 1:500 dilution; mouse anti-REELIN (MBL International, D3513), 1:300 dilution; mouse anti-SATB2 (Abcam, ab51502), 1:100 dilution; rat anti-SST (EMD Millipore MAB354), 1:100 dilution; rabbit anti-TBR1 (Abcam, ab31940), 1:2,000 dilution; chicken anti-TBR2 (EOMES; EMD Millipore, AB15894), 1:1,000 dilution; rabbit anti-ZBTB20 (Sigma-Aldrich, HPA016815), 1:100 dilution. Secondary antibodies used included the following conjugated anti-species-specific IgG antibodies: Alexa Fluor 488-conjugated donkey (Jackson ImmunoResearch), Cy3-conjugated donkey (Jackson ImmunoResearch), and Alexa Fluor 594-conjugated donkey (Jackson ImmunoResearch) at a 1:1,000 dilution, and Alexa Fluor 647-conjugated donkey (Jackson ImmunoResearch) and Alexa Fluor 647-conjugated goat (Invitrogen) at a 1:800 dilution. Hoechst 33342 was included in the secondary antibody mixture at a concentration of 1 *μ* g/ml to stain nuclei. Cell counting was performed by a total of two individuals for the entire study. One of these individuals performed cell counting for each immunostain/panel of immunostains while blinded to experimental groups. Specifically, once both control and experimental images were acquired, IHC image files were assigned a random number by the lab technician and a key was generated. The complete data files were provided to the assigned counter. Once all counts were completed the data was unblinded and statistical analyses were performed. Since Ctrl 2 was added subsequent to all other groups, blinding to genotype was not possible. In this case, the counter was blinded to the identity of the immunostain.

#### Assembloid calcium imaging

The genetically encoded calcium indicator GCaMP6f was introduced into assembloids during days 88–95 through viral transduction using 5 *μ* L of 1:98 × 10^13^ GC mL^− 1^ pGP-AAV-*syn*-jGCaMP7f-WPRE virus^64^ (Addgene, 104488-AAV1). Imaging was conducted 14 to 16 days post-infection utilizing a Leica Stellaris two-photon microscope equipped with a Coherent Chameleon tunable laser. Calcium transients were captured at an excitation wavelength of 920 nm employing a ×25 0.8-NA water-immersion objective (Nikon) at a frame rate of 31 Hz, with a resolution of 512 × 512 pixels and a field of view of 0:5 × 0:5 mm. Recordings were carried out in artificial cerebrospinal fluid (aCSF) supplemented with 10 mM HEPES to maintain a pH between 7.3 and 7.4, in the absence of *O*_2_*/ CO*_2_ perfusion (refer to ‘Local field potential recordings’ section for further details).

Single-neuron calcium imaging data were extracted using a custom MATLAB script that leverages the CaImAn toolbox,^65^ specifically employing the CNMF (Constrained Nonnegative Matrix Factorization) algorithm^66^ for source extraction and spike inference. The data underwent motion correction using the NoRMCorre package.^67^ Components were initialized and iteratively updated, with relevant components selected through correlation and a pre-trained convolutional neural network classifier. The final processed data consisted of ΔF/F traces and inferred raster plots of action potentials.

#### Assembloid local field potential recordings

Organoid recordings were conducted approximately between days 115 and 125. Live organoids were perfused with 250 nM kainic acid in artificial cerebrospinal fluid (aCSF), which consisted of 126 mM NaCl, 10 mM D-glucose, 1.2 mM MgCl_2_, 2 mM CaCl_2_, 5 mM KCl, 1.25 mM NaH_2_PO_4_, 1.5 mM sodium pyruvate, 1 mM L-glutamine, and 26 mM NaHCO_3_, to stimulate oscillatory network activities. The pH was maintained at 7.3–7.4, and aCSF was bubbled with 95% O_2_ and 5% CO_2_. Local field potential (LFP) activity was captured using a patch pipette filled with aCSF, connected to a field amplifier (A-M Systems, 3000). The signal was bandpass filtered between 0.1 and 1,000 Hz through an instrumentation amplifier (Brownlee BP Precision, 210A). Field potentials were digitized at 4,096 Hz using a National Instruments A/D board, interfaced with EVAN, a custom-designed LabVIEW-based software from Thotec. Manual inspection of the raw and power band data was performed to ensure quality, and subsequent analysis was performed using custom procedures developed in MATLAB or Igor Pro.

#### Theta-gamma coupling monophasicity

To analyze the degree of monophasic coupling between theta phase and gamma amplitude in our hippocampal assembloids, human epilepsy patients, and hippocampal circuit simulations, we utilized a custom MATLAB function. This function first defines a set of amplitude frequencies ranging from 25 to 115 Hz and applies a band-pass filter to the local field potential at each frequency band, followed by a Hilbert transform to extract the amplitude envelope. Simultaneously, the data is filtered within the theta band (3–10 Hz) and transformed to obtain the instantaneous phase. The relationship between the amplitude of gamma frequencies and the phase of theta is then computed by averaging the normalized amplitude of gamma at each theta phase bin, divided into 18 bins spanning from −*π* to *π*. This results in a radian modulogram matrix, where each row represents a gamma frequency and each column a theta phase bin. To quantify monophasicity, which indicates whether the amplitude of all gamma frequencies couples predominantly to one phase of theta, the matrix is correlated across all frequency pairs, and the median of these correlation coefficients (excluding the diagonal) is calculated as the monophasicity for each 10-s trial. This process is repeated for all trials in a given dataset, and the overall monophasicity for the organoid or patient is then taken as the median of these values.

#### Instability analysis

To assess the instability of theta rhythms and single neuron spiking dynamics in our organoid models, human data, and simulations, we employed the modified 0–1 chaos test^68–70^ alongside the estimation of the largest Lyapunov exponent for the simulations. The modified 0–1 chaos test involves driving a two-dimensional dynamical system with the recorded time-series data:

(Equation 1) $$\begin{array}{l} p(n+1)=p(n)+\phi (n)\cos(cn),\\ q(n+1)=q(n)+\phi (n)\sin(cn), \end{array}$$

where c is a randomly chosen constant between 0 and 2π. The system’s trajectory, through the computation of the growth rate of the time-averaged mean square displacement of p and q, distinguishes chaotic from periodic dynamics. The mean square displacement is calculated as:

(Equation 2) $$M_{c}(n)=\frac{1}{N}\sum_{j=1}^{N}\left({\left[p_{c}(j+n)-p_{c}(j)\right]}^{2}+{\left[q_{c}(j+n)-q_{c}(j)\right]}^{2}\right)+\sigma \eta_{n},$$

where $\eta_{n}$ is a noise term. We set σ=0.5, based on our prior work.^31^ The correlation coefficient $K_{c}=\mathrm{corr}\left(n,M_{c}(n)\right)$ is calculated for 100 unique values of c, and the median K value is used as the final statistic, with values approaching 1 indicating chaos and values near 0 suggesting periodicity. To estimate the instability of theta waves, the modified 0–1 chaos test was applied to local field potentials (real and simulated) band-pass filtered between 3 and 10 Hz; for estimating the instability of single-neuron spiking, we applied the test directly to the assembloid raster plots (inferred from the calcium imaging data) and to the raster plots of our simulated hippocampal circuit.

For our simulations, we estimated the largest Lyapunov exponent (which can only be estimated for *in silico* models) to directly quantify chaos. The simulations were run twice with a small perturbation in the initial membrane potentials of the neurons in the simulated hippocampal circuit, and the divergence ϵ(t) between these two runs was calculated. The stochastic largest Lyapunov exponent was then estimated from the divergence ϵ(t) between the initial and perturbed runs of the simulation, denoted $Q_{e}^{\left(1\right)}$ and $Q_{e}^{\left(2\right)}$, respectively, was calculated using the cumulative squared differences between their outputs:

(Equation 3) $$\epsilon (t)=\frac{{\left(Q_{e}^{\left(1\right)}(t)-Q_{e}^{\left(2\right)}(t)\right)}^{2}}{\epsilon^{\max}},$$

where $\epsilon^{\max}$ represents the maximum divergence between the two simulation traces:

(Equation 4) $$\epsilon^{\max}={\left(\max\left(Q_{e}^{\left(1\right)}\right)-\min\left(Q_{e}^{\left(2\right)}\right)\right)}^{2}.$$

To estimate the largest Lyapunov exponent Λ, we calculate how the divergence ϵ(t) evolves over time:

(Equation 5) $$\epsilon (t)=\epsilon (0)e^{\Lambda t},$$

where ϵ(0) is the initial divergence at t=0. The Lyapunov exponent Λ can then be inferred from the slope of the natural logarithm of ϵ(t) plotted against time t. Importantly, both runs, $Q_{e}^{\left(1\right)}$ and $Q_{e}^{\left(2\right)}$, were subjected to identical noise conditions to ensure that any observed divergence is due to dynamical differences rather than stochastic variability. The computed slope provides a measure of the stochastic largest Lyapunov exponent, where a positive slope (and thus a positive stochastic largest Lypuanov exponent) indicates chaos/dynamical instability.

#### Hippocampal circuit simulation

The O-LM cell model is adapted from the multicomponent model described in Saraga et al.,^71^ which was initially developed as a multicompartment model. This model was later simplified into a single-compartment model by Tort et al.^33^ This model included fast-spiking basket cells, slow-spiking O-LM cells, and pyramidal cells, each described by Hodgkin-Huxley equations. Building on this simplified model, we have introduced a persistent sodium current (NaP) into the circuit equation for each neuron type, reflecting effects of the specific gain-of-function *p.SCN8A* variants on non-inactivating sodium conductance,^7,34–38^ in order to model the pathophysiology of DEE-13.

The O-LM cell model is described by the current-balance equation:

$$C_{O}\frac{dV_{O}}{dt}=I_{\text{app},O}-I_{\text{Na},O}-I_{\text{NaP},O}-I_{\text{K},O}-I_{\text{L},O}-I_{h,O}-I_{A,O}-I_{\text{syn},O}$$

with $V_{O}$ as the membrane potential, $C_{O}=1.3$ *μF/cm*^2^ as the capacitance, $I_{\text{app},O}$ the applied current, and $I_{\text{syn},O}$ the synaptic current. The leak current $I_{L,O}$ has a conductance of 0:05*mS/cm*^2^ and a reversal potential of − 70*mV*. The currents in this equation are defined as:

$$I_{\text{Na},O}=g_{\text{Na},O}m^{3}h\left(V_{O}-E_{\text{Na},O}\right)\;I_{\text{NaP},O}=g_{\text{NaP},O}m_{p}\left(V_{O}-E_{\text{Na},O}\right)\;I_{\text{K},O}=g_{\text{K},O}n^{4}\left(V_{O}-E_{\text{K},O}\right)\;I_{\text{L},O}=g_{\text{L},O}\left(V_{O}-E_{\text{L},O}\right)\;I_{h,O}=g_{h,O}r\left(V_{O}-E_{h,O}\right)\;I_{A,O}=g_{A,O}ab\left(V_{O}-E_{A,O}\right)$$

where the gating variables are governed by:

$$\alpha_{m}=-0.1\left(V_{O}+38\right)/\left(\exp\left(-0.1\left(V_{O}+38\right)\right)-1\right)\;\beta_{m}=4\exp\left(-\left(V_{O}+65\right)/18\right)\;\tau_{m}=1/\left(\alpha_{m}+\beta_{m}\right)\;m_{\infty }=\;\alpha_{m}\tau_{m}\;\alpha_{h}=\;0.07\exp\left(-\left(V_{O}+63\right)/20\right)\;\beta_{h}=\;1/\left(1+\exp\left(-\left(V_{O}+33\right)/10\right)\right)\;\tau_{h}=\;1/\left(\alpha_{h}+\beta_{h}\right)\;h_{\infty }=\;\alpha_{h}\tau_{h}\;\alpha_{n}=\;0.018\left(V_{O}-25\right)/\left(1-\exp\left(-\left(V_{O}-25\right)/25\right)\right)\;\beta_{n}=\;0.0036\left(V_{O}-35\right)/\left(\exp\left(\left(V_{O}-35\right)/12\right)-1\right)\;\tau_{n}=\;1/\left(\alpha_{n}+\beta_{n}\right)\;n_{\infty }=\;\alpha_{n}\tau_{n}\;\tau_{m_{p}}=1\;m_{\text{fp}}=\;1/\left(1+\exp\left(-\left(V_{O}+52.3\right)/6.8\right)\right)\;\tau_{a}=5\;a_{\infty }=\;1/\left(1+\exp\left(-\left(V_{O}+14\right)/16.6\right)\right)\;\tau_{b}=1/\left(0.000009/\exp\left(\left(V_{O}-26\right)/18.5\right)+0.014/\left(0.2+\exp\left(-\left(V_{O}+70\right)/11\right)\right)\right)\;b_{\infty }=\;1/\left(1+\exp\left(\left(V_{O}+71\right)/7.3\right)\right)\;\tau_{r}\;=\;1/\left(\exp\left(-14.59-0.086V_{O}\right)+\exp\left(-1.87+0.0701V_{O}\right)\right)\;r_{\infty }=\;1/\left(1+\exp\left(\left(V_{O}-v50\right)/10.2\right)\right)$$

The basket cell model, adapting the characteristics of a fast-spiking interneuron as initially described by Wang and Buzsaki^72^ and later refined by Tort et al.,^33^ likewise incorporates a persistent sodium current. It is a single-compartment model described by:

$$C_{I}\frac{dV_{I}}{dt}=I_{\text{app},I}-I_{\text{Na},I}-I_{\text{NaP},I}-I_{\text{K},I}-I_{\text{L},I}-I_{\text{syn},I}$$

where $V_{I}$ denotes the membrane potential, $C_{I}=1\mu \;F/cm^{2}$ indicates the membrane capacitance, $I_{\text{app},I}$ is the applied current, $I_{\text{syn},I}$ is the total synaptic current, and $I_{L,I}=g_{L,I}\left(V_{I}-E_{L,I}\right)$ with a conductance of 0:1*mS=cm*^2^ and a reversal potential of − 65*mV*. All currents are measured in *μA=cm*^2^. The currents in this equation are defined as:

$$I_{\text{Na},I}=g_{\text{Na},I}m^{3}h\left(V_{I}-E_{\text{Na},I}\right)\;I_{\text{NaP},I}=g_{\text{NaP},I}m_{p}\left(V_{I}-E_{\text{Na},I}\right)\;I_{\text{K},I}=g_{\text{K},I}n^{4}\left(V_{I}-E_{\text{K},I}\right)\;I_{\text{L},I}=g_{\text{L},I}\left(V_{I}-E_{\text{L},I}\right)$$

where gating variables and their kinetics are given by:

$$\alpha_{m}=-0.1\left(V_{l}+35\right)/\left(\exp\left(-0.1\left(V_{l}+35\right)\right)-1\right)\;\beta_{m}=4\exp\left(-\left(V_{l}+60\right)/18\right)\;\tau_{m}=\;1/\left(\alpha_{m}+\beta_{m}\right)\;m_{\infty }=\;\alpha_{m}\tau_{m}\;\alpha_{h}=\;0.07\exp\left(-\left(V_{l}+58\right)/20\right)\;\beta_{h}=1/\left(1+\exp\left(-0.1\left(V_{l}+28\right)\right)\right)\;\tau_{h}=\;1/\left(\alpha_{h}+\beta_{h}\right)\;h_{\infty }=\;\alpha_{h}\tau_{h}\;\alpha_{n}=0.01\left(V_{1}+34\right)/\left(1-\exp\left(-0.1\left(V_{1}+34\right)\right)\right)\;\beta_{n}=0.125\exp\left(-\left(V_{I}+44\right)/80\right)\;\tau_{n}=\;1/\left(\alpha_{n}+\beta_{n}\right)\;n_{\infty }=\;\alpha_{n}\tau_{n}\;\tau_{m_{p}}=1\;m_{\text{fp}}=1/\left(1+\exp\left(-\left(V_{l}+52.3\right)/6.8\right)\right)$$

Finally, the pyramidal cell model is based on the multicompartmental framework first described by Migliore et al.,^73^ which was subsequently adapted into a more computationally efficient model by Tort et al.^33^ In our current work, we have added a persistent sodium current (NaP) to this model, reflecting a deeper understanding of the pyramidal neurons’ intrinsic properties that affect their long-term excitability and connectivity within neural circuits.

The pyramidal cell model includes basal dendrites (200 *μm* length, 2 *μm* diameter), a soma (20 *μm* length, 20 *μm* diameter), andt hree apical dendrites (each 150 *μm* length, 2 *μm* diameter). Cytoplasmatic resistivity (*Ra*) is set at 150 Ω⋅*cm*. The model for each compartment *k* is governed by:

$$C_{E,k}\frac{dV_{E,k}}{dt}=I_{\text{app},Ek}-I_{\text{Na},Ek}-I_{\text{NaP},Ek}-I_{\text{K},Ek}-I_{\text{L},Ek}-I_{\text{syn},Ek}+I_{\text{conn},Ek}$$

where the currents are defined as:

$$I_{\text{Na},Ek}=g_{\text{Na},E}m^{3}h\left(V_{E,k}-E_{\text{Na},E}\right)\;I_{\text{NaP},Ek}=g_{\text{NaP},E}m_{p}\left(V_{E,k}-E_{\text{Na},E}\right)\;I_{\text{K},Ek}=g_{\text{K},E}n^{4}\left(V_{E,k}-E_{\text{K},E}\right)\;I_{\text{L},Ek}=g_{\text{L},E}\left({\overset{`}{V}}_{E,k}-E_{\text{L},E}\right)$$

and the gating variables and their kinetics are given by:

$$\alpha_{m}=0.32\left(\frac{V_{E,k}+54}{1-\exp\left(-\frac{V_{E,k}+54}{4}\right)}\right)$$

$$\beta_{m}=0.28\left(\frac{V_{E,k}+27}{\exp\left(\frac{V_{E,k}+27}{5}\right)-1}\right)$$

$$\tau_{m}=\;\frac{1}{\alpha_{m}+\beta_{m}}$$

$$m_{\infty }=\;\frac{\alpha_{m}}{\alpha_{m}+\beta_{m}}$$

$$\alpha_{h}=0.128\exp\left(-\frac{V_{E,k}+50}{18}\right)$$

$$\beta_{h}=\frac{4}{1+\exp\left(-\frac{V_{E,k}+27}{5}\right)}$$

$$\tau_{h}=\;\frac{1}{\alpha_{h}+\beta_{h}}$$

$$h_{\infty }=\;\frac{\alpha_{h}}{1+\beta_{h}}$$

$$\alpha_{n}=0.032\left(\frac{V_{E,k}+52}{1-\exp\left(-\frac{V_{E,k}+52}{5}\right)}\right)$$

$$\beta_{n}=0.5\exp\left(-\frac{V_{E,k}+57}{40}\right)$$

$$\tau_{n}=\;\frac{1}{\alpha_{n}+\beta_{n}}$$

$$n_{\infty }=\;\frac{\alpha_{n}}{1+\beta_{n}}$$

$$\tau_{m_{p}}=15$$

$$m_{\text{fp}}=\frac{1}{1+\exp\left(-\frac{V_{E,k}+52.3}{6.8}\right)}$$

To simulate the electrophysiological characteristics of iCtrl Hc+GE assembloids, specifically recreating the observed monophasic theta-gamma coupling, we utilized a genetic optimization approach. This process involved adjusting the parameters of the above-described hippocampal neural network model incorporating O-LM cells, basket cells, and pyramidal cells. The genetic algorithm optimized several key parameters to achieve a close match with the observed electrophysiological data. These parameters included the strength and noise level of the driving currents applied to these cells (measured in picoamperes, pA), the strengths of transient sodium, persistent sodium, and potassium currents across all cell types, and the connectivity between all pairs of cell types (e.g., O-LM to O-LM, O-LM to pyramidal, etc.). To simulate Mut Hc+GE assembloids, we again employed a genetic algorithm, with the iCtrl simulation parameters as the starting point, and optimized the cell numbers and strength of persistent sodium currents to recreate the aberrant theta rhythmicity and theta-gamma coupling, but preserved theta power, of the Mut Hc+GE assembloids.

#### Sample preparation for single-nuclei RNA sequencing

For each assembloid condition from the R1872 > L *p.SCN8A* (iCtrl Cx+GE day 120, Mut P1 Cx+GE day 120, iCtrl Hc+GE day 120, Mut P1 Hc+GE day 120, iCtrl Hc+GE day 84, Mut P1 Hc+GE day 84), three batches of assembloids, each from a different differentiation, were pooled together into a single tube to be dissociated. In this manner, variability across differentiations was accounted for. Overall, between 3 and 11 assembloids per condition (across all batches) underwent the dissociation/sample preparation steps. All sample preparation steps were performed on dry ice or ice to minimize degradation effects from sample thawing or humidity. Tissue chunks were transferred to pre-cooled Dounce tissue grinder (Millipore Sigma, #D9063), and homogenized in RNase-free conditions with a light detergent (0.001% Triton100X/1% BSA/RNase Inhibitor (0.2 *U*/ *μL*)/0.5X Protease Inhibitor/1 *μM* DTT/PBS). The homogenate was filtered through a 40 *μm* micro-cell strainer (Diagnocine, #FNK-HT-AMS-04002) to remove large material and centrifuged at 1000g for 8 min at 4 ^◦^ C to pellet away from debris. The nuclei pellet was washed two more times with PBS/1% BSA/RNase Inhibitor and spun down at 500g for 5 min. The pellet was then suspended in wash buffer for quality assessment and concentration measurement. Nuclei quality was evaluated based on shape, membrane integrity and chromatin structure under 40× magnification, and counted using a countess II machine. The isolated nuclei were then sent to the UCLA Technology Center for Genomics & Bioinformatics to construct expression libraries using the 10x Genomics Chromium Single Cell 3’ (v3 chemistry) system. The libraries aimed to recover 10K nuclei using a recommended concentration range. RNA sequencing on the NovaSeqS4 Illumina achieved an acceptable read depth for each condition (between 27,541–78,121 mean read pairs/cell for 4,613–9,813 cells per condition). All samples achieved >96% genes mapped to the genome.

#### Single-nuclei RNA sequencing analysis

The FASTQ file sequencing data for each assembloid condition (iCtrl Cx+GE day 120, Mut P1 Cx+GE day 120, iCtrl Hc+GE day 120, Mut P1 Hc+GE day 120, iCtrl Hc+GE day 84, Mut P1 Hc+GE day 84) were processed using the Cell Ranger 7.1.0 pipeline to generate count data. These were subsequently processed using custom Python v3.11.8 based scripts that implemented the Scanpy v1.9.8 single cell analysis package.^53^ Briefly, genes that were expressed in less than 30 cells across all conditions were filtered out. Next, thresholds for total RNA counts and unique feature counts were determined for each condition via manual inspection in order to filter out multiplets and low quality cells. Manual thresholds were used as these counts were not normally or symmetrically distributed. Total RNA count thresholds typically ranged between 1000 and 15000 counts per cell, with 1000–8000 unique features per cell. Cells with >1% mitochondrial RNA were also filtered out. Scrublet^54^ was used to ensure adequate removal of doublets. Next, cell cycle analysis of S phase and G2/M phase gene markers (such as *MCM5*, *PCNA*, and *CDK1*, *NUSAP1*, respectively) was performed to confirm that relatively few S and G2/M phase cells were present in the data. After these initial quality control steps, the remaining high-quality cells were integrated across all 6 conditions using the single cell variational inference package scVI v1.1.2^55^ (using 2 hidden layers with 128 nodes per layer, a latent space of 30 dimensions, and gene likelihood modeled by a zero-inflated negative binomial distribution). Nearest neighbors were calculated using a local neighborhood size of 15 with distance metric based on the Pearson correlation coefficient given previously reported optimal performance^74^ prior to UMAP projection. Visual inspection was subsequently performed to verify reasonable integration across batches. Leiden clustering using multiple resolutions in a highly iterative manner was performed to achieve gross cell type labeling based on canonical expression markers (e.g.,^75–78^ see upregulated gene markers in Table S5). ExN and INs were subsequently isolated and each was independently reintegrated across batches using the prior method to perform subtype clustering and labeling. ExN and IN cluster labeling (Figures S5A and S5B, respectively) utilized previously reported canonical expression markers.^79–86^

After cell labeling, a scANVI integration step^87^ was utilized to generate the UMAP plots in Figures 1, 7, and S6 in order to maximize biological conservation during batch integration. DGE analysis was performed using Scanpy’s built-in functions (Wilcoxon method with tie-correct and Bonferroni correction) given their previously reported optimal performance.^88,89^ DGE significance testing utilized a threshold of p-adjusted <0.05 and log-fold change of >0.5 and was performed individually for each cell type (where at least 30 cells were present across all conditions) to focus the DGE analysis on within-cell-type differences (as cell-type differences were analyzed independently). To check for pathological changes through DGE analysis, significantly downregulated genes were compared to the list of genes that, when dysfunctional, are associated with epilepsy (CUI: C0014544) and intellectual disability (CUI: C3714756) using DisGenNet (https://www.disgenet.org/search ).^44^ Significantly upregulated genes from DGE analysis were also compared to the list of genes associated with glutamatergic/excitatory functions. Gene ontology analysis was performed for all upregulated and downregulated genes using the Gene Set Enrichment Analysis package in Python (GSEApy)^56^ with the human *GO_Biological_Process_2021* dataset and a significance cutoff of p-adjusted <0.05. Super-clustering was performed using the GO-Figure package.^90^ As noted previously, the co-expression network module identification in Figure S7 was performed using the hdWGCNA package.^45^

### QUANTIFICATION AND STATISTICAL ANALYSIS

Graph generation and statistical analyses were performed either on GraphPad Prism 10 or MATLAB software. In cases where there were repeated measurements within a single sample (e.g., repeated calcium indicator measurements from the same organoid) a linear mixed effects model was used to determine statistical differences. All other samples were subjected to Shapiro–Wilk and Kolmogorov–Smirnov normality testing. Non-normal samples were analyzed by a one- or two-tailed Wilcoxon rank-sum test or Kruskal–Wallis test (and if warranted) followed by a Dunn’s multiple-comparison test. A Wilcoxon signed-rank test was used for paired data (patient hippocampal electrode recordings). Normally distributed data were analyzed by a two-tailed Student’s t-test or ANOVA with post hoc Tukey’s multiple-comparison test unless specified in the figure legend. Sample sizes were not pre-determined but are comparable to prior publications. Organoids were generated from hiPSCs in batches of 96 organoids per plating and subjected to visual inspection with each media change (q2–3 days) and more detailed visual quality control checks at the following timepoints: day 6, 18, and 35. Of the samples that passed these quality control checks, individual organoids were randomly selected for experiments. To determine variability of assembloid sizes, 120D iCtrl and Mut P1 Cx+GE and HC + GE were imaged in brightfield adjacent to a ruler. The images with ruler were exported to ImageJ where exact assembloid perimeters were quantified (Figure S1G). All cell counting was performed by an individual blinded to experimental group (or immunostain) and data were unblinded only after all counts were completed for a specific immunostain. Other than cell counting, which was performed as detailed above, no other data collection or analysis was performed in a blinded fashion. No data points were excluded from analysis for any reason. Bar graphs indicate the mean and standard deviation of the data, and all individual data points are plotted to demonstrate the full distribution of values. Figure legends specify sample numbers (n) and exact *p* values for all statistical tests, and include all other relevant details. Each *n* value represents an independent experiment performed on an assembloid or organoid generated from an independent hiPSC differentiation.

## Supplementary Material

Figures S1–S7 and Table S1

Video S5

Video S6

Table S2

Table S3

Table S4

Table S5

Video S1

Video S2

Video S3

Video S4

Supplemental information can be found online at https://doi.org/10.1016/j.celrep.2025.116217.

## Figures and Tables

**Table:** KEY RESOURCES TABLE

REAGENT or RESOURCE	SOURCE	IDENTIFIER

Antibodies		

rabbit anti-CAMK2	ProteinTech	Cat # 13730–1-AP; RRID: AB_2070320
rat anti-CTIP2	Abcam	Cat # ab18465; RRID: AB_2064130
rabbit anti-DLX1	generous gift of S. K. Lee and J. Lee	None
guinea pig anti-DLX2	generous gift 929 of K. Yoshikawa and H. Shinagawa	None
mouse anti-GAD65	BD Biosciences	Cat # 559931; RRID: AB_397380
rabbit anti-GRIK4	Invitrogen	Cat # PA5–111717; RRID: AB_2857127
mouse anti-Nkx2.1	Novocastra	Cat # NCL-L-TTF-1; RRID: AB_564042
goat anti-NRP2	R&D Systems	Cat # AF2215; RRID: AB_2155371
rabbit anti-OLIG2	EMD Millipore	Cat # AB9610; RRID: AB_570666
rabbit anti-PAX6	MBL International	Cat # PD022; RRID: AB_1520876
mouse anti-PROX1	EMD Millipore	Cat # MAB5654; RRID: AB_2170714
rabbit anti-PV	Abcam	Cat # ab11427; RRID: AB_298032
mouse anti-REELIN	MBL International	Cat # D3513; RRID: AB_2815002
mouse anti-SATB2	Abcam	Cat # ab51502; RRID: AB_882455
rat anti-SST	EMD Millipore	Cat # MAB354; RRID: AB_2255365
rabbit anti-TBR1	Abcam	Cat # ab31940; RRID: AB_2200219
chicken anti-TBR2/EOMES	EMD Millipore	Cat # AB15894; RRID: AB_10615604
rabbit anti-ZBTB20	Sigma Aldrich	Cat # HPA016815; RRID: AB_1858947
Alexa Fluor 400-conjugated donkey	Jackson ImmunoResearch	
Cy3-conjugated donkey	Jackson ImmunoResearch	
Alexa Fluor 594-conjugated donkey	Jackson ImmunoResearch	
Alexa Fluor 647-conjugated donkey	Jackson ImmunoResearch	

Bacterial and virus strains		

pGP-AAV1-*syn*-jGCaMP7f-WPRE	Addgene	104488-AAV1
pAAV1-CAG-tdTomato	Addgene	59462-AAV1

Deposited data		

Expression data from this study	Gene Expression Omnibus	GEO: GSE281622

Experimental models: Cell lines		

iPSC: SCN8A Mut R1872 > L (P1)	University of Michigan, Dr. Jack Parent	*p.SCN8A* Mut (P1)
iPSC: SCN8A iCtrl R1872 > L (P1)	University of Michigan, Dr. Jack Parent	*p.SCN8A* iCtrl (P1)
iPSC: SCN8A Mut V1592 > L (P2)	University of Michigan, Dr. Jack Parent	*p.SCN8A* Mut (P2)
iPSC: iCtrl MAPT Tau R406W	Tau Consortium, Dr. Celeste Karch	Ctrl 2

Software and algorithms		

Original code used for data analysis in this pape	https://github.com/samarasinghela	
SCANPY v1.9.8 single cell analysis package	Wolf et al.^53^	
CELL RANGER 7.1.0	10x Genomics	
SCRUBLET	Wolock et al.^54^	
single cell variational inference package 1114 SCVI v1.1.2	Lopez et al.^55^	
GSEAPY	Fang et al.^56^	
HDWGCNA	Morabito et al.^45^	
GRAPHPAD PRISM 10	Dotmatics	
MATLAB	Mathworks	

## Highlights

- Cortical assembloids with SCN8A mutations exhibit marked network hyperexcitability
- Hippocampal assembloids show theta-gamma coupling deficits, mirroring patient recordings
- Computational modeling predicts selective O-LM interneuron loss in the hippocampus
- scRNAseq and IHC reveal region-specific neuronal identity changes in DEE-13 assembloids
