# Supplementary material for: Cortical versus hippocampal network dysfunction in a human brain assembloid model of epilepsy and intellectual disability
Source: Cell Rep. Author manuscript; Available in PMC 2025 Dec 3. (PMC12674601; doi:10.1016/j.celrep.2025.116217)
Supplement: Figures S1–S7 and Table S1 [file NIHMS2113394-supplement-Figures_S1_S7_and_Table_S1.pdf]

Cell Reports, Volume *44*

Supplemental information

Cortical versus hippocampal network dysfunction in a human brain assembloid model of epilepsy and intellectual disability

Colin M. McCrimmon, Daniel Toker, Marie Pahos, Qing Cao, Kevin Lozano, Jack J. Lin, Jack M. Parent, Andrew Tidball, Jie Zheng, László Molnár, Istvan Mody, Bennett G. Novitch, and Ranmal A. Samarasinghe

Supplemental Figures

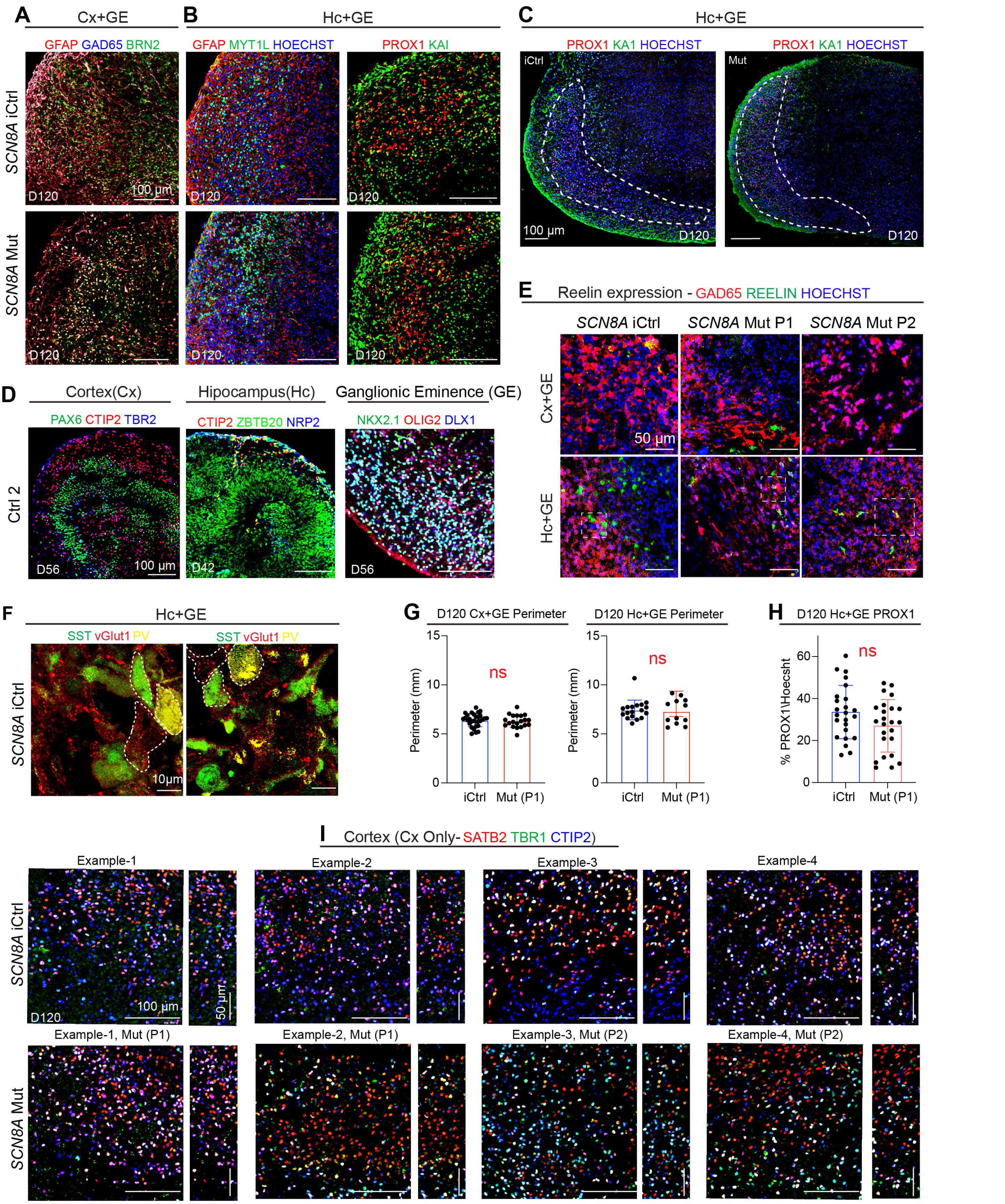

Figure S1. Cx+GE and Hc+GE Immunohistochemistry Demonstrates the Presence of Expected Cell Types, Conserved Assembloid Sizes, and Layered Organization, related to Figures 1, 5, 7. A, Immunohistochemical analysis of iCtrl and Mut Cx+GE fusion organoids at D120 reveals the presence of glial (GFAP), GABAergic interneuron (GAD65), and the neuronal marker (BRN2). **B**, Immunohistochemical analysis of iCtrl and Mut Hc+GE at D120 reveals the presence of GFAP and the nuclear-localized

neuronal marker MYTL1 co-localized with Hoechst, as well as distinct dentate granule (PROX1) and CA3-like (KA1) regions. **C**, Identical replicates of D120 Hc+GE shown in Figure 1D showing PROX1+ dentate granule cells distinct from KA1+ CA3-like regions, here including Hoechst to delineate the boundary of the nucleus. **D**, Immunohistochemical analysis of Ctrl 2 iPSC derived Cx, Hc, and GE organoids at the specified ages demonstrates appropriate layered cellular architecture comparable to the isogenic control (shown in Figure 1). **E**, Identical replicates of Figure 7I and 7J demonstrating GAD65+/Reelin+ inhibitory interneurons in Hc+GE, but not in Cx+GE (see Figure 7 for quantification). Here showing Hoechst in order to delineate the cell nucleus. **F**, Representative examples of high resolution immunohistochemical analysis of D120 iCtrl Hc+GE assembloids revealing multiple examples of the tripartite cellular ensemble consisting of PV-SST-Pyramidal cells (the latter immunostained by the excitatory pre-synaptic marker Vglut1). Similar tripartite ensembles were observed in 41 out 46 regions of interest (approximately 89 percent) taken from four imaged sections representing two independently generated assembloids. **G**, Box plots of the perimeter of D120 Cx+GE and Hc+GE iCtrl and Mut P1 assembloids. Each dot represents an independently generated organoid (with group mean $\pm$ SD shown), the data represent at least 4 independent differentiations/batches, and demonstrate relatively low within/between batch variance for each genotype and no significant differences between the variants. **H**, Box plots of PROX1 expressing cells as a percentage of total Hoechst+ cells in D120 iCtrl and Mut P1 reveals no significant differences. Each dot represent a section (with group mean $\pm$ SD shown), n=6 independent organoids (differentiations) per genotype, 4 sections per organoid, linear mixed effects model, p=0.2408. **I**, Independently generated examples demonstrating the layered architecture of D120 cortical organoids. The images demonstrate the broadly consistent pattern of SATB2+ upper/superficial layer neurons, CTIP2+ deep layer neurons, and TBR1+ intermediate progenitors across genotypes, as well as the inherent sample-to-sample variation.

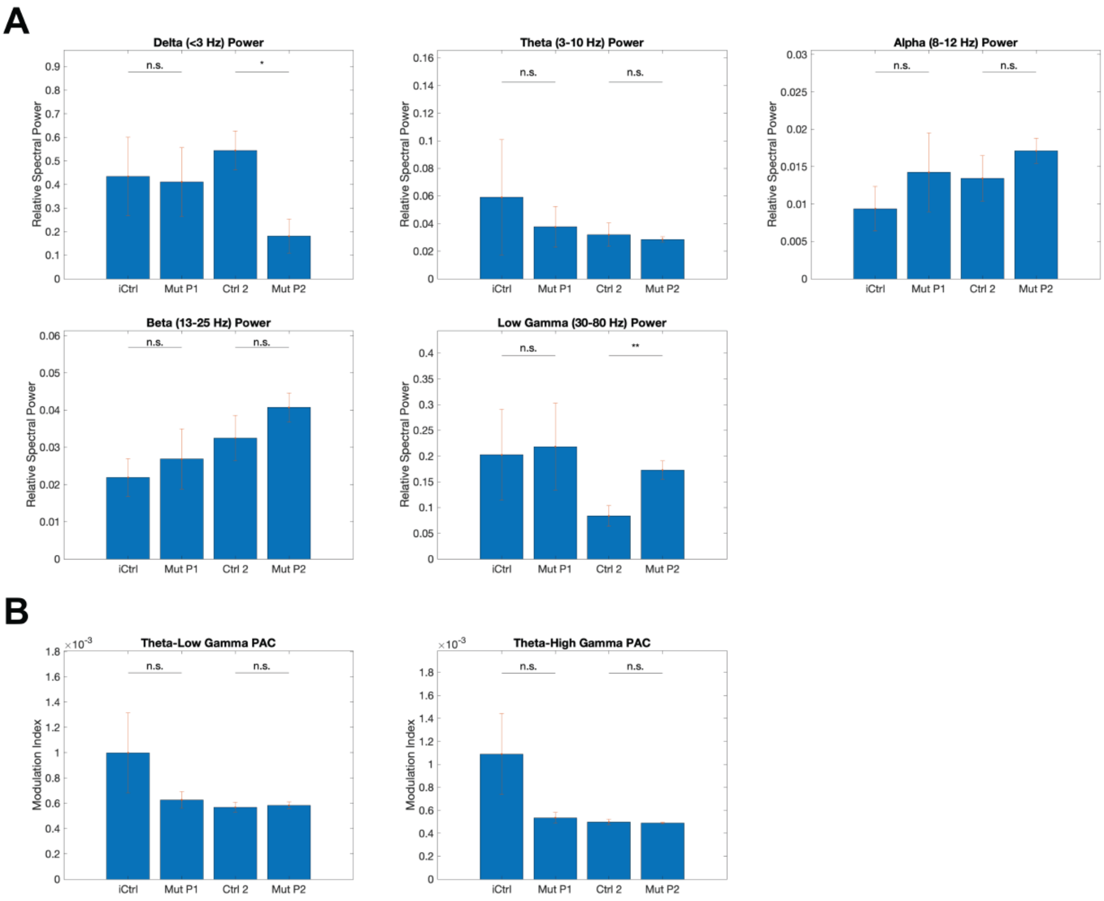

Figure S2. Relative Spectral Power Across Different Frequency Bands and Strength of Theta-Gamma Phase-Amplitude Coupling in Hc+GE Assembloids, related to Figures 3, 4. A, Spectral power was calculated using Welch's power spectral density estimate. Each datapoint represents the median relative spectral power in a given frequency band for a single assembloid, across all 10-second trials available for that assembloid. The mean ± SD of these datapoints are shown. There were no consistent differences in the relative spectral power across canonical frequency bands (delta, theta, alpha, beta, and low gamma) between iCtrl and Mut assembloids. Notably, the only significant differences observed were an increase in relative alpha (8-12 Hz) and beta (13-25 Hz) power in Mut Patient 2 Hc+GE assembloids compared to controls (two-tailed Wilcoxon rank-sum tests). These findings indicate that the disruption of monophasic theta-gamma phase-amplitude coupling in both Mut Patient 1 and Mut Patient 2 Hc+GE assembloids, as shown in Figure 4, is likely not attributable to changes in the overall power of oscillatory rhythms in the local field potentials recorded from these assembloids. ns= $p>0.05$, *= $p<0.05$, **= $p<0.01$. **B**, We used the modulation index to measure the strength of coupling between the phase of theta (3-10 Hz) rhythms and the amplitude of both low gamma (30-80 Hz) and high gamma (80-160 Hz) rhythms in the Hc+GE assembloids presented in Figure 4E-G. The mean ± SD for each assembloid type are shown. Notably, there were no significant differences between the iCtrl and Mut assembloids (two-tailed Wilcoxon rank-sum tests).

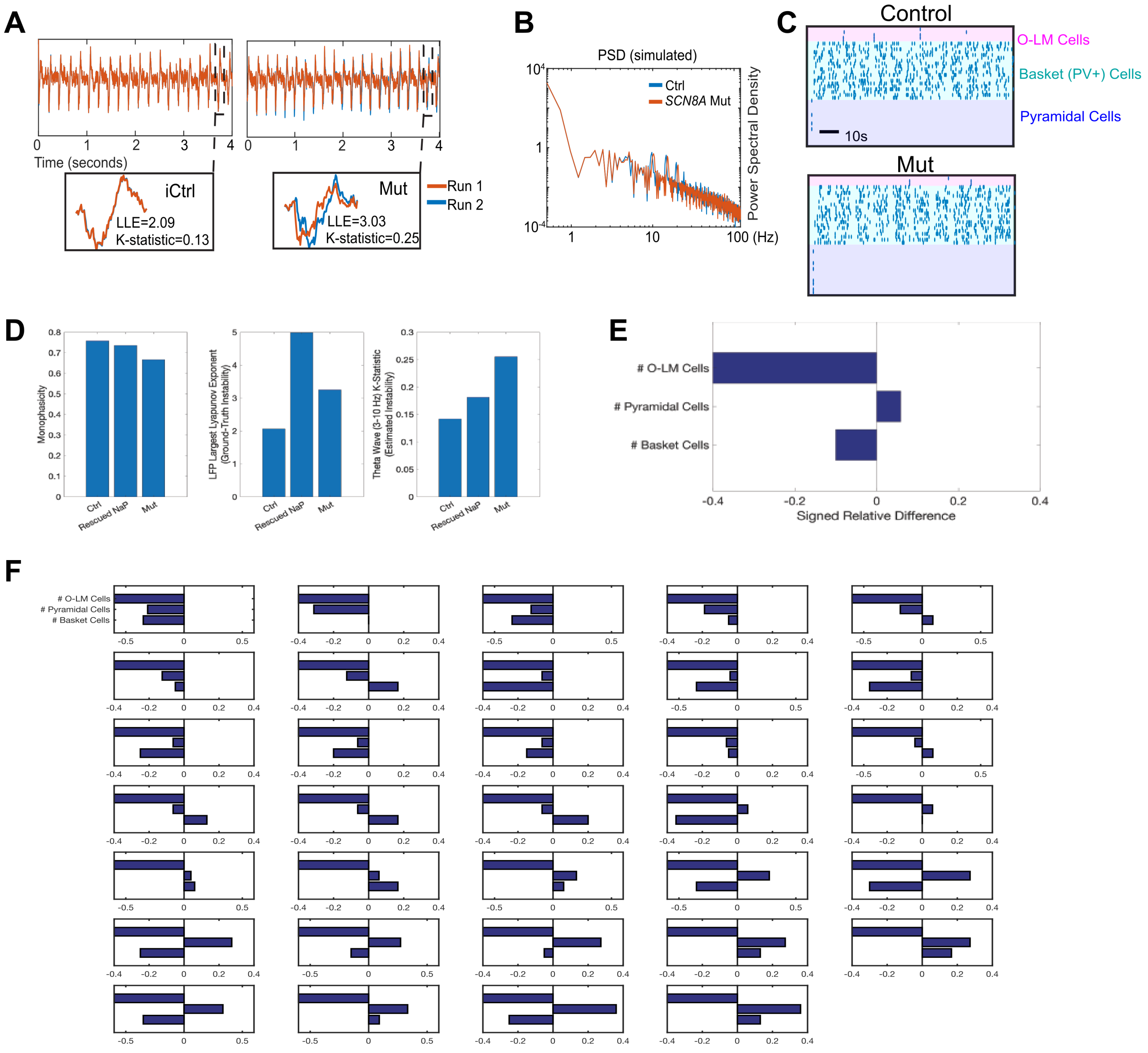

Figure S3. Modeling hippocampal circuit alterations and instability in DEE-13, related to Figure 5. **A**, Perturbation analysis of simulated theta oscillations in iCtrl and Mut networks. A small initial perturbation (blue trace) produces minimal divergence from the original oscillation (orange) in iCtrl, but increasingly diverges in Mut, indicating heightened dynamical instability. This is quantified by higher largest Lyapunov exponents (LLE) and K-statistics in Mut. **B**, Power spectral density of simulated local field potentials from iCtrl and Mut networks, showing comparable theta and gamma power across conditions. **C**, Simulated spike rasters for iCtrl and Mut networks. Mut simulations exhibit more irregular firing patterns across pyramidal, basket, and O-LM cell populations. **D**, Theta–gamma coupling, LLE, and K-statistic metrics comparing iCtrl, Mut, and a "rescued Mut" simulation in which persistent sodium current levels were restored to iCtrl values while preserving the Mut circuit composition. Although sodium current normalization slightly improved network dynamics, it did not fully restore rhythmic coupling or reduce instability, suggesting that correcting ion channel dysfunction alone may be insufficient to restore hippocampal network function in DEE-13. **E**, Harmonic mean of cell-type ratios across all alternative simulated CA3 networks that reproduced the Mut electrophysiological phenotype (reduced theta–gamma coupling, increased instability, and preserved theta/gamma power). On average, these models showed reduced O-LM cells, increased pyramidal cells, and slightly reduced basket cells—paralleling the machine learning–optimized circuit and aligning with IHC observations in Patient 2. **F**, Cell-type configurations across all successful simulations that reproduced the Mut phenotype. The only consistent feature across all configurations was reduced O-LM cell number, while pyramidal and basket cell numbers varied—suggesting that O-LM cell loss may be a core driver of network dysfunction in SCN8A-related DEE-13.

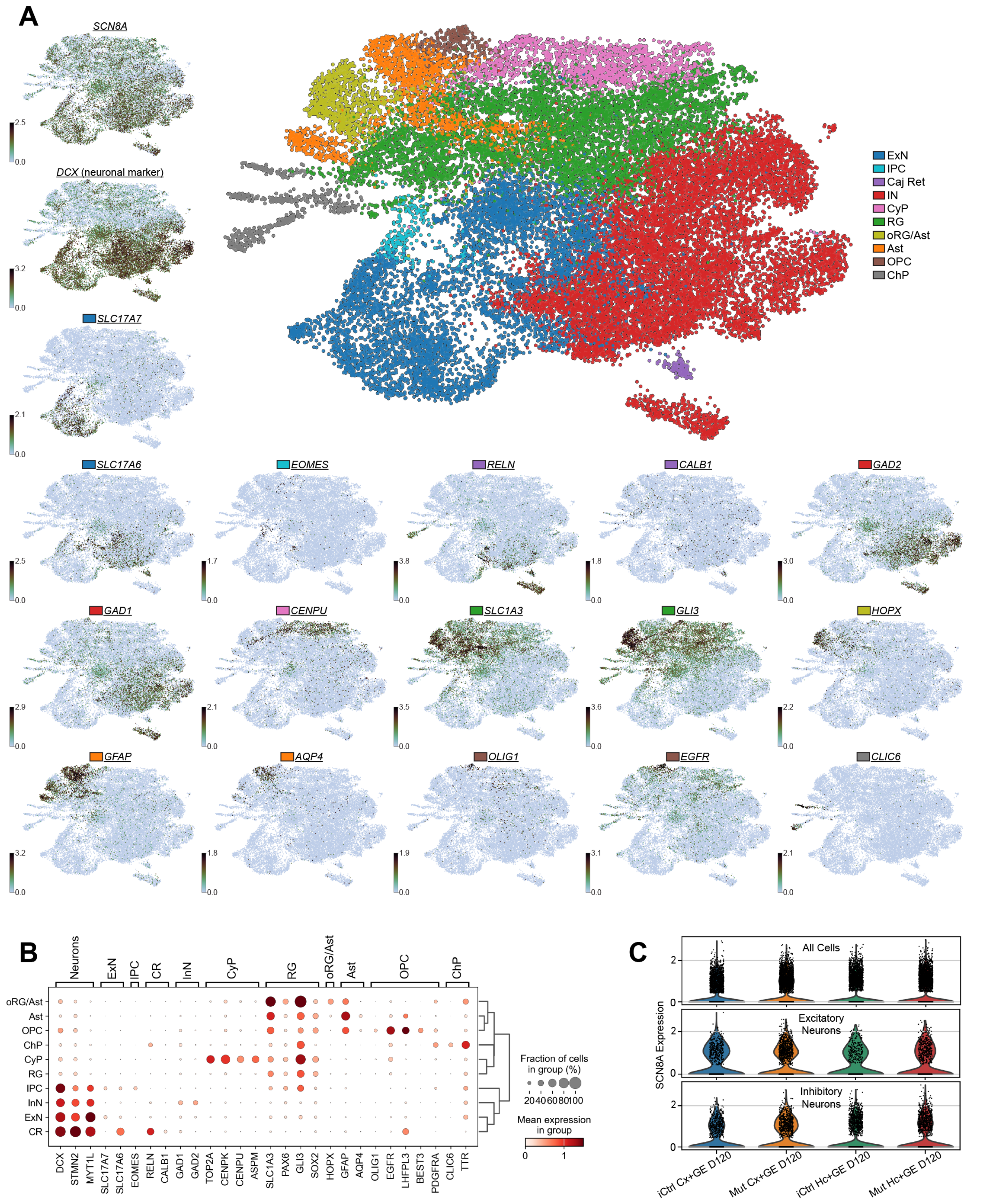

Figure S4. Cortical and Hippocampal Assembloids Contain Diverse Cell Types with Canonical Expression Patterns, related to Figures 1, 7. A, 2D UMAP of all cells from both iCtrl and Mut P1 Cx+GE and Hc+GE assembloids integrated together that demonstrates diffuse expression of *SCN8A* across cell and assembloid types. The cells were well separated into neuronal (*DCX*-expressing) and non-neuronal types. These include *SLC17A6-*/*SLC17A7*-expressing excitatory neurons (ExN), *EOMES*-expressing intermediate progenitors (IPC), *RELN-*/*CALB1*-expressing Cajal-Retzius (Caj Ret), *GAD2-*/*GAD1*-expressing inhibitory neurons (IN), *CENPU*-expressing cycling progenitors (CyP), *SLC1A3-*/*GLI3*-expressing radial glia (RG), *HOPX*-expressing outer radial glia (oRG) that overlapped partially with *GFAP*-expressing astrocytes (Ast) in a separate oRG/Ast co-cluster (in addition

to distinct Ast with more mature expression markers such as *AQP4*), *OLIG1*-/*EGFR*-expressing oligodendrocyte precursors (OPC), and (*CLIC6*)-expressing choroid plexus. **B**, Dotplot of canonical expression markers consistent with cell type labels in (A). **C**, Violin plots demonstrating similar levels of *SCN8A* expression across Day 120 iCtrl and Mut Cx+GE and Hc+GE batches for all cells (top), excitatory neurons only (middle), and inhibitory neurons only (bottom) suggesting against a pure cell-autonomous effect of *p.SCN8A*.

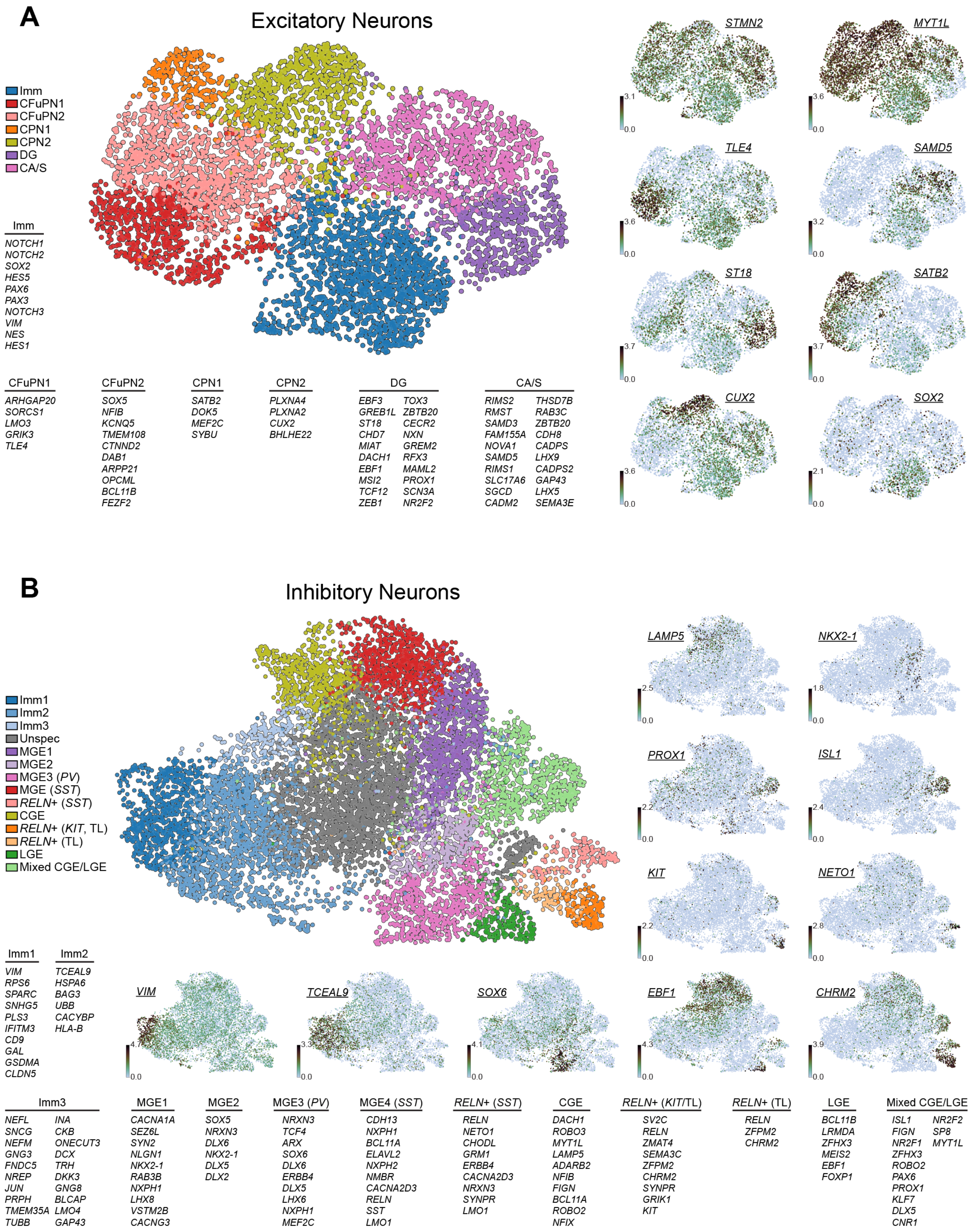

Figure S5. Excitatory and Inhibitory Neuron Subtypes in Cortical and Hippocampal Assembloids, related to Figures 1, 7. A, 2D UMAP of excitatory neuron subtypes from both iCtrl and Mut P1 Cx+GE and Hc+GE assembloids showing well-defined clusters. These correspond to Imm immature excitatory neurons with relatively increased immature markers (but still strongly expressing *STMN2*, *MYT1L*, and *DCX* from Figure S4, indicative of neuronal fate), CFuPN1/2 corticofugal projection neurons (*TLE4*, *BCL11B*, *FEZF2*), CPN1/2 callosal projection neurons (*SATB2*, *CUX2*, *BHLHE22* as well as more mature markers, e.g. *SYBU*), DG dentate granule-like neurons (canonical dentate gyrus and hippocampal markers, e.g. *PROX1*, *ST18*, *ZBTB20*),

and CA/S cornu ammonis/subiculum-like neurons (*LHX9*, *THSD7B*, *CADPS*/*CADPS2*, *SLC17A6*). **B**, 2D UMAP of inhibitory neuron (IN) subtypes from both iCtrl and Mut P1 Cx+GE and Hc+GE assembloids showing diverse clusters. These correspond to: immature INs at various stages (Imm1 = *VIM*, *SPARC*, Imm2 = *TCEAL9*, *BAG3*, Imm3 = *NEFL*, *SEFM*, *ONECUT3*), unspecified (Unspec) INs that lacked expression markers definitively corresponding to specific known IN subtypes, multiple populations of medial ganglionic eminence-like (MGE) INs including less differentiated MGE1 and MGE2 (*NKX2-1*, *LHX8*, *DLX6*), MGE3 (*PV*) putative parvalbumin INs (*SOX6*, *ERBB4*, *MEF2C*), (*SST*) MGE4 putative somatostatin INs (*SST*, *NMBR*). CGE caudal ganglionic eminence-like (*LAMP5*, *ADARB2*) and LGE lateral ganglionic eminence-like (*EBF1*, *LRMDA*, *MEIS2*) INs were present. One separate Mixed LGE/CGE cluster expressing genes canonical for both LGE and CGE INs (*ISL1*, *PAX6* as well as *PROX1*, *NR2F2*) was present. Additionally, there was a distinct super-cluster of *RELN*+ INs that included: *SST* MGE (*NETO1*, *CHODL*, *GRM1*), *KIT*/TL CGE INs (expressing *KIT* and the trilaminar interneuron marker *CHRM2*), and TL CGE INs (*CHRM2* only). All listed genes are differentially expressed in that cluster (p-adj$<$0.05) and are listed in order of significance.

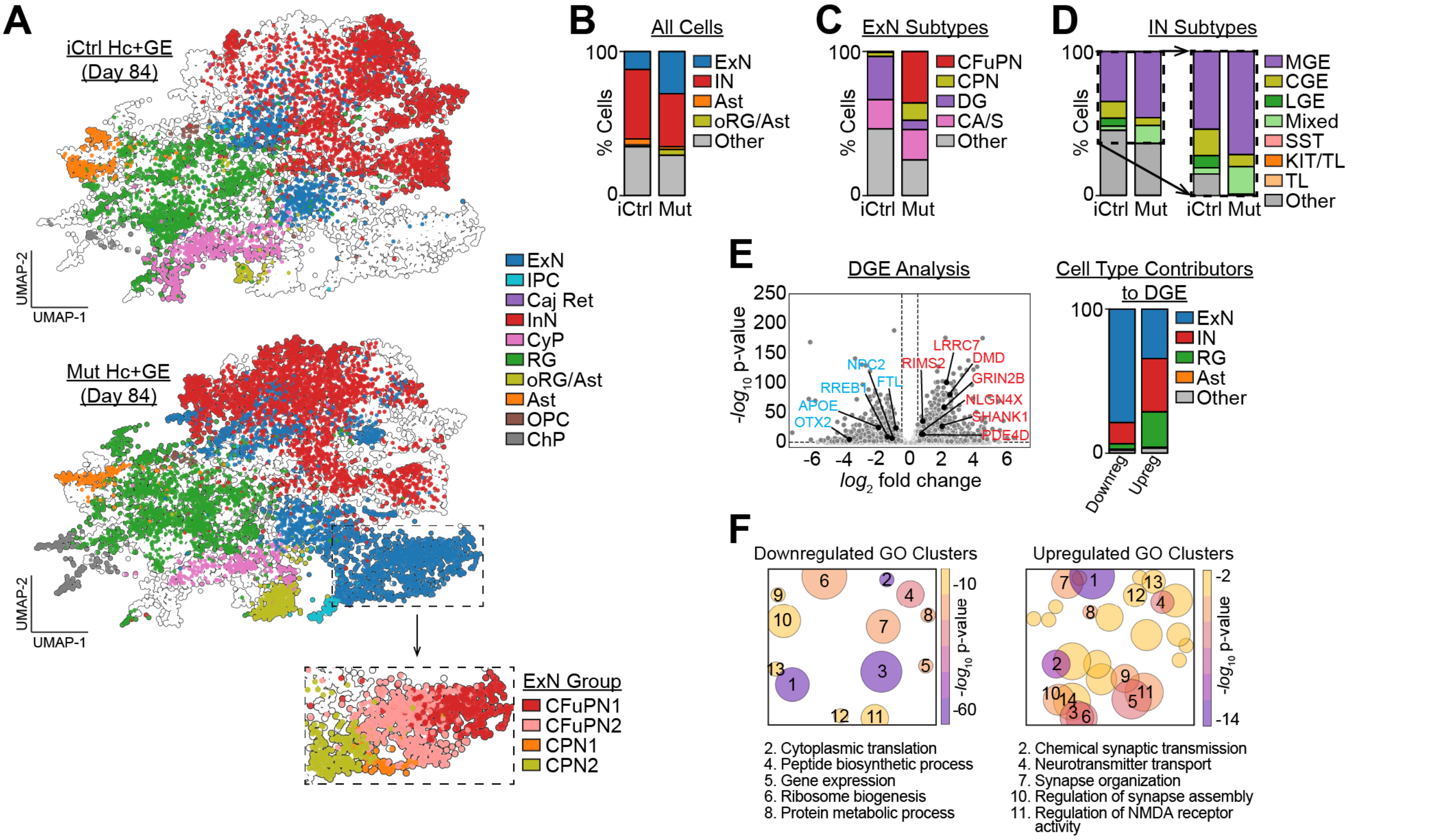

Figure S6. Early Effects of DEE-13 on Circuit Composition and Gene Expression in Hippocampal Assembloids at Day 84 Show Similarities and Differences Versus Day 120 Assembloids, related to Figures 6, 7. A, 2D UMAP of cells from iCtrl and Mut P1 Hc+GE at day 84 showing diverse cell types including excitatory neurons (ExN), intermediate progenitors (IPC), Cajal-Retzius (Caj Ret), inhibitory neurons (IN), cycling progenitors (CyP), radial glia (RG), outer radial glia (oRG), astrocytes (Ast), oligodendrocyte precursors (OPC), choroid plexus (ChP). There was an increase in cortex-like ExN subtypes, i.e. corticofugal (CFuPN) and callosal (CPN) projection neurons, in the MutP1 Hc+GE assembloids (see zoomed in box of ExN group). **B**, Mut P1 Hc+GE assembloids show increased numbers of ExNs and decreased INs compared to iCtrl, similar to the Day 120 Hc+GE assembloids. Likewise, there is a reduction in Ast and increase in the oRG/Ast co-cluster, which was also present in the Day 120 Hc+GE assembloids. **C**, Mut P1 Hc+GE assembloids had an increase in cortex-like CFuPN and CPN ExNs compared to iCtrl and a decrease in hippocampal dentate granule-like (DG) ExNs, mirroring what was seen at Day 120 although without a substantial reduction in cornu ammonis/subiculum-like (CA/S) ExNs. **D**, There was a mild reduction of medial ganglionic eminence (MGE)-like INs in Mut P1 Hc+GE compared to iCtrl without a large change in the overall caudal or lateral ganglioninc eminence-like (CGE, LGE respectively) INs. No *RELN*+ INs (including SST, KIT/TL, and TL) were present in either iCtrl or Mut P1 Day 84 Hc+GE. **E**, Differential gene analysis volcano plot with a select subset of (red) upregulated genes in Mut P1 assembloids (predominantly in ExNs, INs, RG) that are involved in glutamatergic pathways and a subset of (blue) downregulated genes (predominantly in ExNs) that are associated with epilepsy and intellectual disability when their expression is reduced (from DisGeNET). These genes are shown in relative proportion compared to those in Figure 7K-L. **F**, Gene ontology (GO) analysis using all differentially expressed genes and super-clustering of terms demonstrated a upregulation of genes involved in synaptic function in Day 84 hippocampal Mut P1 assembloids compared to iCtrl as well as a broad downregulation of pathways involved in basic cellular viability/housekeeping tasks (e.g. translation, gene expression, metabolism); this was overall similar to the trend observed in Day 120 assembloids (Figure 7M-N) although at Day 120 the upregulated synaptic genes were more strongly involved in glutamatergic activity. Larger circles correspond to a higher number of GO terms.

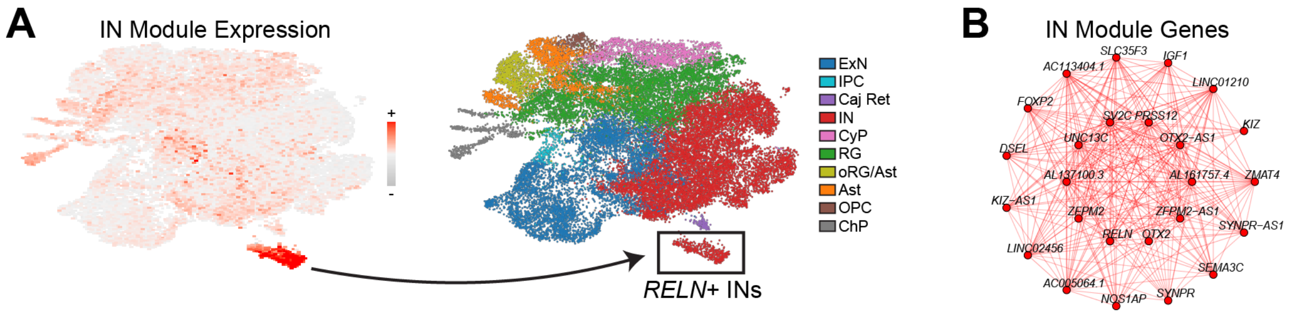

Figure S7. Network Analysis Identifies Co-expressed Genes that are Specific for *RELN*+ INs, related to Figure 7. A Network co-expression analysis (hdWGCNA) of inhibitory neurons across all batches revealed one module corresponding largely to the *RELN*+ IN cluster present in D120 iCtrl Hc+GE from Figure 7E,H. **B** The co-expressed genes in this network overlapped substantially with our DEG results from Figure 7L. Congruent with the clinical phenotype of *p.SCN8A*/DEE13, many of these genes are known to be associated with epilepsy and intellectual disability when dysfunctional (or depleted such as in Mut Hc+GE).

Supplemental Tables

Patient Number	Age	Gender	Epileptic Side
1	44	Male	Left
2	33	Female	Right
3	25	Female	Right
4	31	Female	Left
5	50	Male	Right
6	39	Male	Right
7	31	Male	Right
8	37	Female	Left

Table S1. Temporal Lobe Epilepsy Patient Demographics, related to Figure 4. All temporal lobe epilepsy patients whose hippocampal field potentials were analyzed in Figure 4I-M received bilateral intracranial depth electrode implantations. However, in each patient, epileptic activity was confined to only one hippocampus, with a healthy contralateral hippocampus.
